# Mechanistic modeling of light-induced chemotactic infiltration of bacteria into leaf stomata

**DOI:** 10.1371/journal.pcbi.1007841

**Published:** 2020-05-08

**Authors:** Mohsen Ranjbaran, Mina Solhtalab, Ashim K. Datta

**Affiliations:** 1 Department of Biological and Environmental Engineering, College of Agriculture and Life Sciences, Cornell University, Ithaca, New York, United States of America; 2 Department of Civil and Environmental Engineering, McCormick School of Engineering and Applied Science, Northwestern University, Evanston, Illinois, United States of America; University of Illinois at Urbana-Champaign, UNITED STATES

## Abstract

Light is one of the factors that can play a role in bacterial infiltration into leafy greens by keeping stomata open and providing photosynthetic products for microorganisms. We model chemotactic transport of bacteria within a leaf tissue in response to photosynthesis occurring within plant mesophyll. The model includes transport of carbon dioxide, oxygen, bicarbonate, sucrose/glucose, bacteria, and autoinducer-2 within the leaf tissue. Biological processes of carbon fixation in chloroplasts, and respiration in mitochondria of the plant cells, as well as motility, chemotaxis, nutrient consumption and communication in the bacterial community are considered. We show that presence of light is enough to boost bacterial chemotaxis through the stomatal opening and toward photosynthetic products within the leaf tissue. Bacterial chemotactic ability is a major player in infiltration, and plant stomatal defense in closing the stomata as a perception of microbe-associated molecular patterns is an effective way to inhibit the infiltration.

## Author summary

Exposure to light can trigger photosynthesis in plant leaves, such as leafy-greens, and increase concentrations of photosynthetic products, such as glucose, within the leaf tissue. Bacteria existing at the leaf surfaces may respond to the available photosynthetic products and migrate into the leaf tissue by chemotaxis toward nutrient concentration gradients. Once the bacteria are inside the leaf tissue, they cannot be washed away, presenting a risk to the consumer. Here, a physics-based model for this light-driven infiltration is presented. This mechanistic model couples transport of bacteria and nutrients, and photosynthesis within a leaf tissue around one stomatal opening. The model shows that the ability of bacteria to transport via chemotaxis is a major factor in infiltration. A moderate intensity light is sufficient to promote chemotactic infiltration of bacteria on a leaf surface into its interior. Infiltration is enhanced in the presence of blue, white and red lights, and for a larger stomatal aperture.

## Introduction

Several bacteria, such as *Escherichia coli* and *Salmonella enterica*, are able to attach the microstructure at the surface of plant leaves, such as trichomes, stomata and grooves [1], and localize at sites that are not accessible for wash water and sanitizers. The bacteria are also able to infiltrate into available openings at the leaf surface, such as stomata, cuts and wounds, to reach tens of micrometer depths below the leaf epidermis [2]. This infiltration presents a risk to public health by causing serious foodborne outbreaks as consumption of raw leafy greens has been on the rise over the past decades [3].

Light is one of the driving forces that can promote infiltration of pathogenic bacteria into plant leaves. Incubation of *S. enterica* serovar Typhimurium on iceberg lettuce leaves in the light led to association of bacteria near open stomata and infiltration into the leaf tissue. However, a dark condition caused a scattered attachment pattern at the leaf surface and a poor stomatal infiltration [2]. Nutrients, such as glucose and sucrose, produced by photosynthetically active cells in the leaf tissue during light exposure are attractive for bacteria that may be initially present at the leaf surface [4]. Opening of the stomata in light brings up an opportunity for bacteria to transport via chemotaxis toward the gradients of nutrients into the leaf interior. Many plants have evolved stomatal defense machinery to close the stomata upon perception of bacterial surface structures, known as microbe-associated molecular patterns (MAMPs) [5]. However, it is not always successful and some human pathogens were shown to penetrate the leaf interior through a process involved with chemotaxis and motility [2].

The process of light-driven infiltration is complex involving plant photosynthesis and respiration, and transport of gases, nutrients and bacteria, that are all interconnected. These processes are affected by various factors including leaf properties, bacterial features and environmental conditions. A deeper understanding of such a complex system can be obtained through development of a mechanistic model that integrates all the relevant biological processes with the physics of transport. A mechanistic model can provide a comprehensive understanding of how various factors contribute to the overall process. Such a model, by its nature, can isolate the effect of a particular factor that is difficult to obtain through experiment alone. While models exist for individual elements of this complex system such as for bacterial chemotaxis [6] and growth [7], and plant photosynthesis [8], they have never been combined.

### Objectives

The objectives of this manuscript are to: 1) develop a model for chemotactic and motile transport of bacteria through an open stoma into the leaf interior toward the concentration gradients of photosynthetic products of glucose and oxygen, 2) couple this model with a model of photosynthesis and respiration, and related multicomponent transport of gases and sugar inside a leaf as a porous medium, 3) validate the models against literature and experimental data for photosynthetic products generated and the amount of bacterial infiltration into the leaf interior, and 4) identify the most important parameters and quantify their relative contributions to light-driven bacterial infiltration.

## Results and discussion

The model includes various biological factors related to plant leaf and bacteria, and they are briefly discussed in S1 Text. These information include an overview of a typical leaf cross section (Fig Aa in S1 Text), various organelles within plant cells (Fig Ab in S1 Text), photosynthetic machinery in chloroplasts (Fig Ac in S1 Text), mechanisms of sugar transport across plasma membrane of the plant cells, underlying pathways leading to bacterial motility (Fig Ba in S1 Text), chemotaxis (Fig Bb in S1 Text), synthesis of quorum sensing signalling molecules of autoinducer-2 (AI-2) (Fig Bc in S1 Text), and bacterial glucose uptake (Fig Bd in S1 Text).

**Fig 1:**
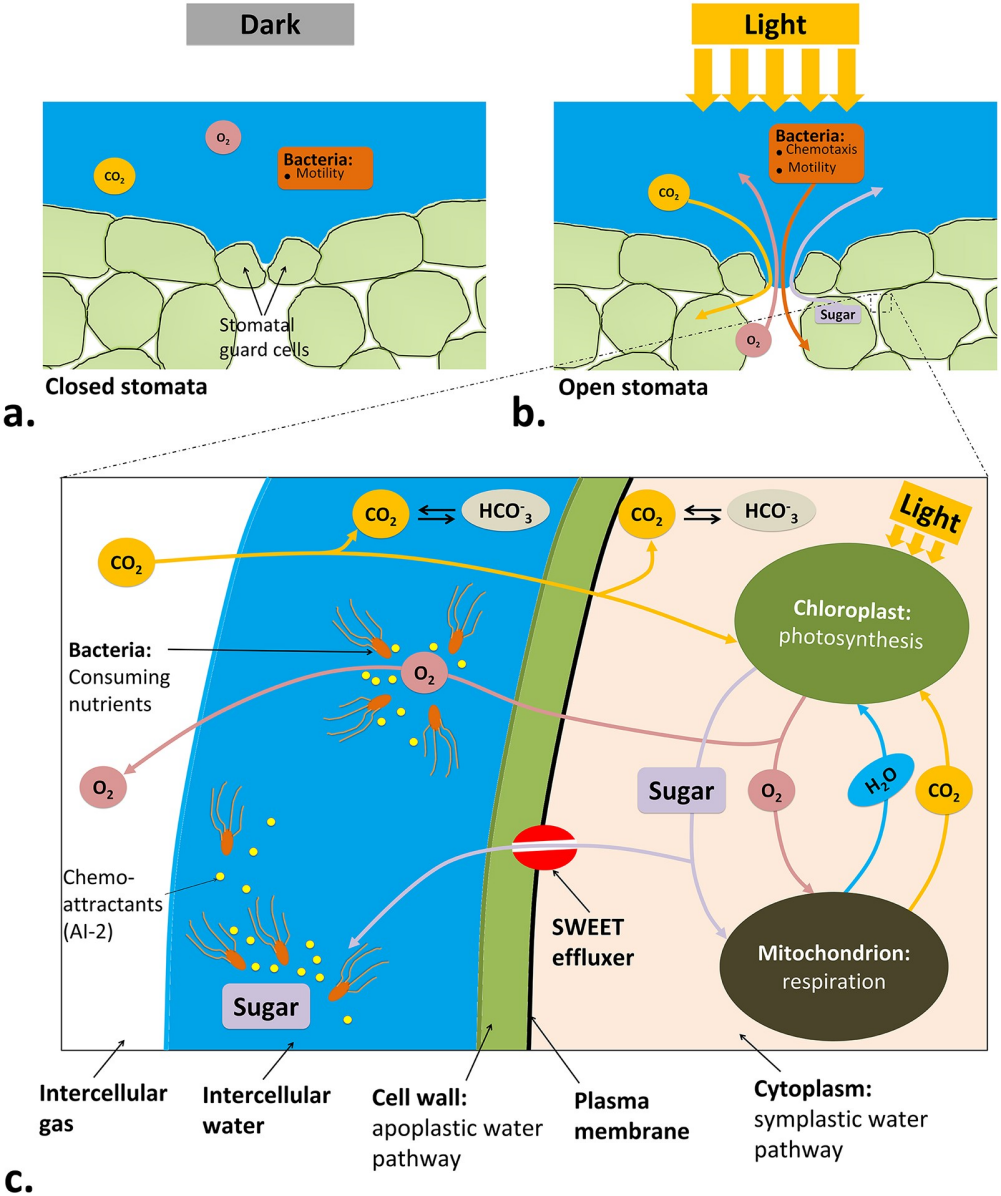
Physical schematic focusing on a stomatal opening under a) dark and b) light conditions. c) A closeup look at the underlying pathways within a leaf tissue that result in light-induced bacterial infiltration into the leaf.

**Fig 2:**
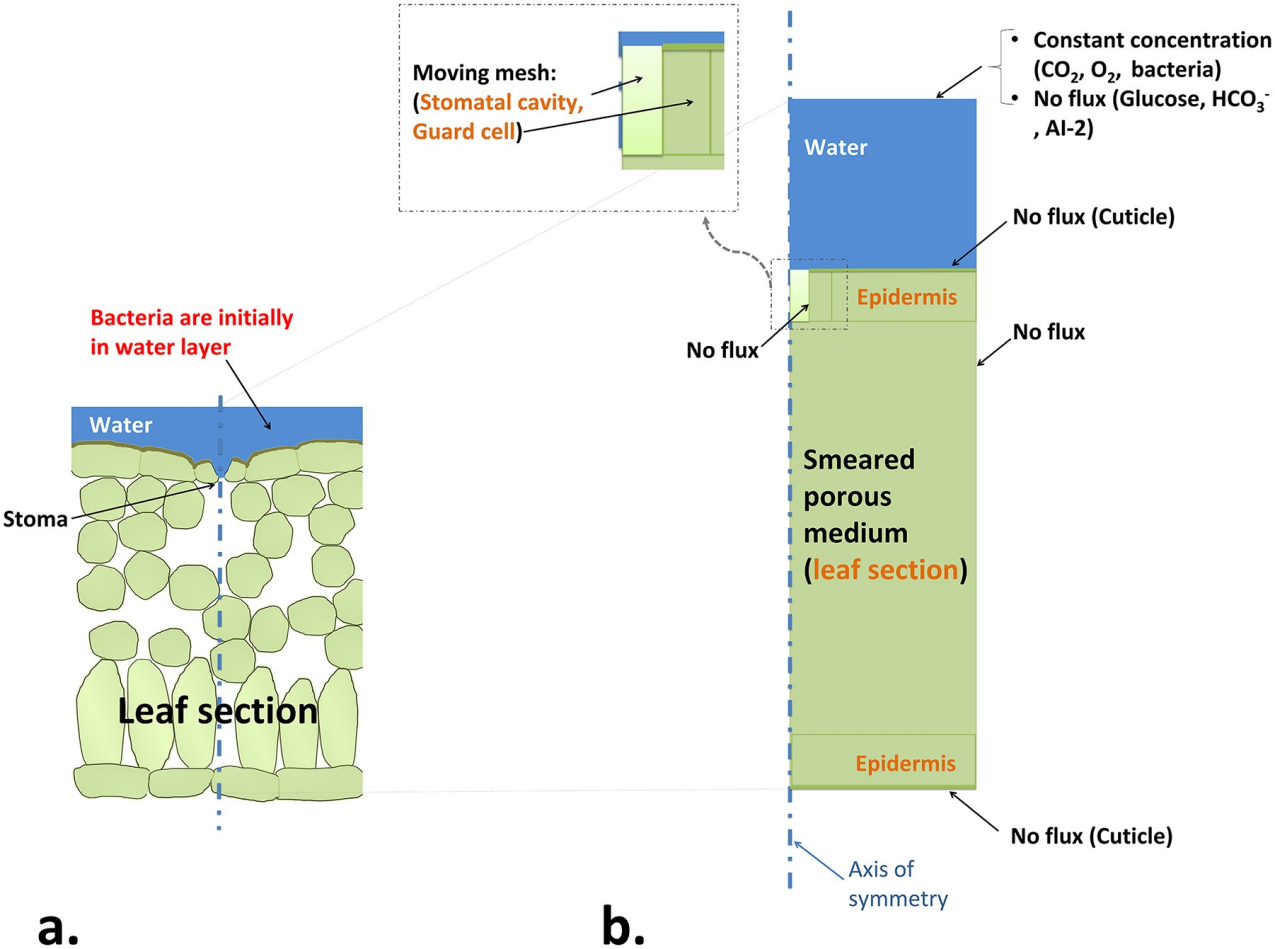
a) Physical schematic of a leaf section around one stomatal opening, and a water layer at the surface. b) Model schematic showing a 2D-axisymmetric computational solution domain (a leaf section and a water layer at its surface). The boundary conditions are also shown. Note that this image is provided as a schematic and does not imply the exact dimensions of the different domains used in the simulations. The entire leaf section (see Fig A in S1 Text for more details), including mesophyll, epidermis layers, guard cell and stomatal cavity, is part of the porous media zone. See Fig C in S1 Text for an overview of all involved species in the model, their transport modes and how they are interconnected via source terms.

Fig 1 shows a schematic of the physical processes in light-driven active internalization of chemotactic bacteria into leaves through a stomatal opening. This study considers the leaf surface to be covered by a layer of water containing bacteria. In the absence of light, stomatal guard cells remain closed (Fig 1a). Shining light on the leaf triggers opening of the stomatal pore (Fig 1b) [9] which enhances the gas exchange into or out of the leaf tissue. Exposure to light also induces carbon fixation in the photosynthetic cells, including mesophyll cells and stomatal guard cells, leading to synthesis of various types of sugars as well as oxygen. These nutrients attract bacteria, initially being within the water film at the leaf surface, to reach the stomatal opening and infiltrate the leaf [2]. Fig 1c provides a closer look at major pathways that occur inside a mesophyll cell during light exposure, leading to production of photosynthetic products, their transport across plasma membrane, and consumption by bacteria in the apoplast. Carbon dioxide (CO_2_) can transport into the leaf tissue and diffuse in intercellular water, the cell wall and plasma membrane to reach the chloroplast. Meanwhile, some CO_2_ may be hydrated into bicarbonate (${\text{HCO}}_{3}^{-}$). Sugar (which is assumed to be glucose/sucrose) and O_2_ are produced due to photosynthesis in the chloroplast, a portion of it being reused in mitochondrion to regenerate CO_2_ and water. The excessive O_2_ diffuses out of the cell into intercellular water (where it can attract bacteria) and gas. In apoplastic loader plants like spinach, the excessive sugar is transported to the apoplast to be loaded into the phloem [10]. Here, it is assumed that sugar efflux through SWEET transporter proteins happens at the mesophyll plasma membrane [11]. So, sugar can diffuse into the intercellular water and be consumed by bacteria (Fig 1c). Bacteria transport via motility and chemotaxis toward nutrients and chemoattractants such as autoinducer-2 (AI-2), starting from the layer of water outside to the intercellular space of the leaf interior. A computational schematic showing details of the solution domain around one stomatal opening is illustrated in Fig 2 and summarized in the Materials and methods section.

Below, the model predictions and validations are presented for the rate of CO_2_ fixation and the amount of sugar production during photosynthesis. This is followed by studying the effects of light intensity and wavelength, bacterial transport mode, leaf side and stomatal defense on the amount of infiltration. After a sensitivity analysis on the most important parameters in the model, the primary and secondary factors affecting the infiltration are highlighted.

### CO_2_ fixation and nutrients production

**Fig 3:**
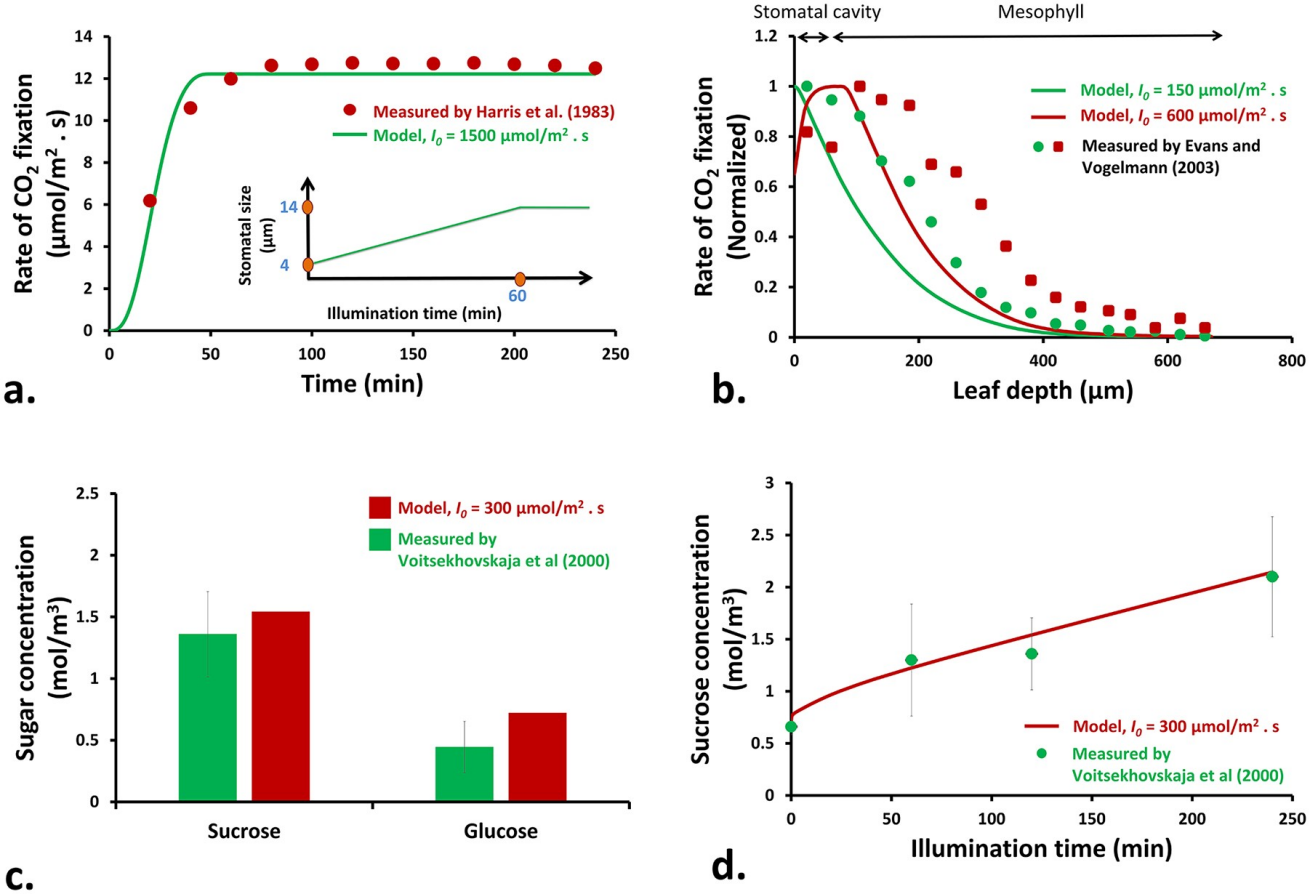
a) Variation of the rate of CO_2_ fixation within spinach tissue at white light intensity of 1500 *μ*mol/m^2^ · s and atmospheric CO_2_ level of 0.013 mol/m^3^. The predicted results are compared with experimental data of Harris et al. [12]. b) Variation of the normalized rates of CO_2_ fixation within the leaf tissue during exposure to blue light. The trends are compared with normalized trends of flourescent emissions within spinach tissue [15]. c) Total amount of apoplastic sucrose and glucose concentrations in spinach leaves after 120 min illumination with white light intensity of 300 *μ*mol/m^2^ · s [18], and d) predicted and experimental [18] variation of apoplastic sucrose concentration in spinach leaves during 4 h of illumination with a white light intensity of 300 *μ*mol/m^2^ · s.

To show the performance of the model of photosynthesis [8] (known as FvCB model) when coupled to the main model (Fig. C in S1 Text), the predicted rate of CO_2_ assimilation in spinach leaves was compared with the experimental measurements of Harris et al. [12] (Fig 3a). In computations, the leaf was initially assumed to be in a dark condition and stoma was partially closed. As illumination occurred, the stoma was gradually opened by a moving mesh approach with a prescribed speed of 1.4 nm/s that is analogous to the rate of stomatal opening in 1 h (by assuming a constant rate). As CO_2_ diffuses into the leaf tissue during illumination, the rate of CO_2_ fixation increases until it equilibrates with the exposed conditions. A step function was used here to help predictions better match the experimental condition reported in Harris et al. [12]. The results are in agreement with predicted and experimental values of the rate of photosynthesis in tomato leaves [13].

To validate model predictions for distribution of the rate of CO_2_ fixation within the leaf tissue, the model predictions were qualitatively compared with experimental profiles of the rate of ^14^C fixation within spinach leaves, during exposure to blue light, obtained from measurement of chlorophyll fluorescence [14, 15] (Fig 3b). As can be seen, the rate of carbon fixation declined toward the leaf depth since the light was absorbed by the chlorophyll pigments within the chloroplasts. It remained higher through the leaf tissue when a higher level of light intensity was exposed to the leaf because high-density chlorophyll pigments (Fig. Da in S1 Text) within the depth of the leaf can absorb more light. Similar distributions of CO_2_ fixation within the leaf tissue can be obtained for red, green and white lights [15–17].

After sugar was synthesized within the plant cells, it was effluxed by SWEET transporter proteins located at the plasma membrane into the apoplast (Fig 1c). This resulted in an increase in the concentration of apoplastic sugar while the leaf was illuminated. The apoplastic sucrose and glucose in spinach leaves, with inhibited phloem transport, were measured by Voitsekhovskaja et al. [18]. Their measured experimental data were compared with model predictions for synthesis of sucrose and glucose during illumination with 300 *μ*mol/m^2^ · s, as shown in Fig 3c and 3d. The data presented in Fig 3 are for the condition that bacteria were absent.

### Bacterial infiltration

Many of the identified fresh produce associated disease outbreaks in the US were caused by *E. coli* [19, 20]. Therefore, the model was validated based on the experimental data obtained from infiltration of *E. coli* into plant leaves.

#### Effect of light exposure on infiltration

**Fig 4:**
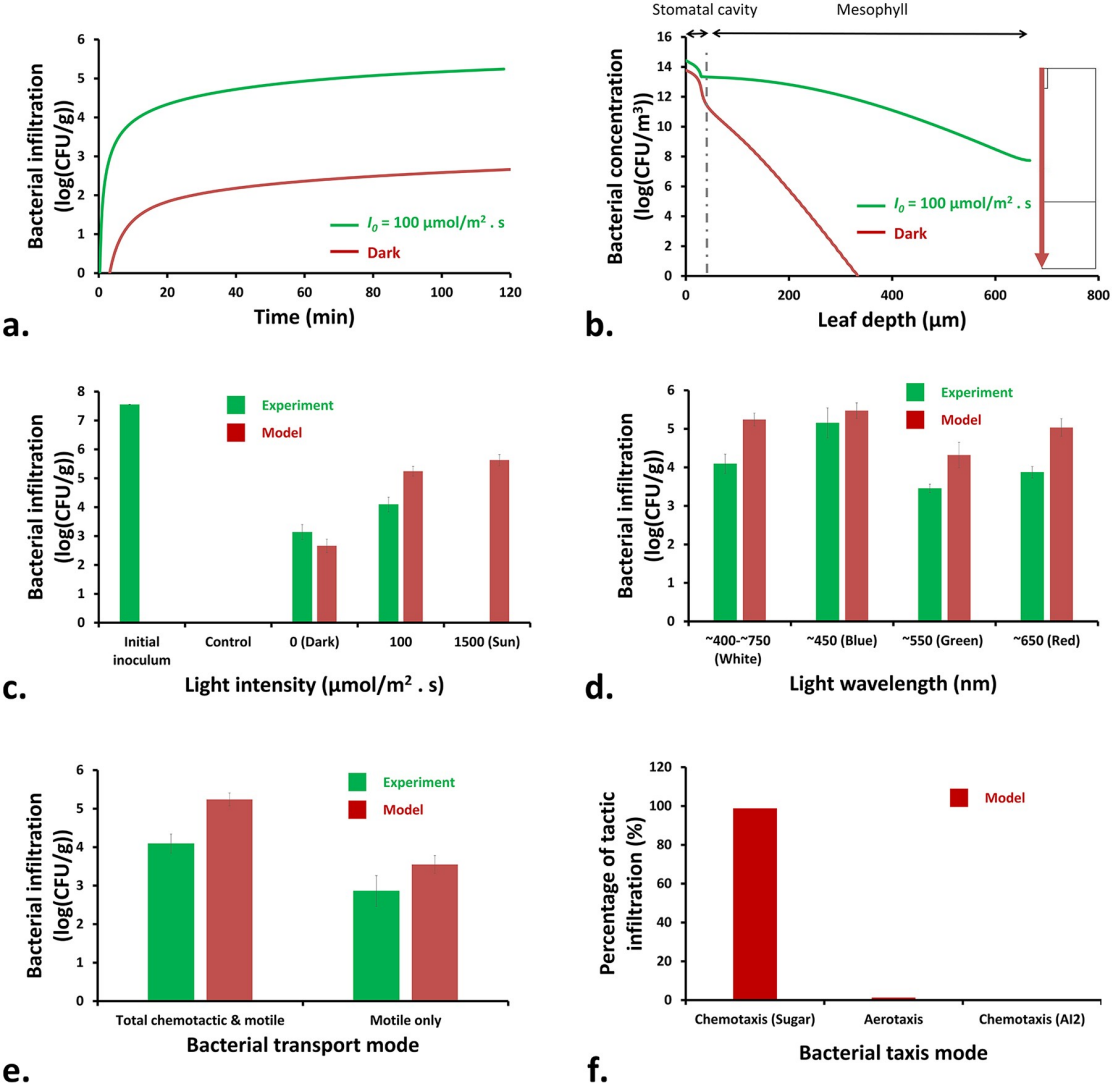
Variations of the a) amount and b) depth of bacterial infiltration into the leaf tissue under white light intensity of 100 *μ*mol/m^2^ · s and dark conditions. Total amount of bacterial infiltration for different levels of c) light (white) intensities and d) wavelengths (at 100 *μ*mol/m^2^ · s) are shown. e) Total amount of bacterial infiltration for different modes of bacterial migration. f) Relative contributions of the three different tactic migration modes. Error bars on the experimental data represent the standard deviation. For the predicted data, the error bars show the effect of 30% change in the stomatal aperture on the amount of bacterial infiltration. See Fig. G and Fig. I-N (S1 Text) for supporting information related to the experimental data presented here.

Typical variations of the amount of bacterial infiltration into the leaf tissue during light and dark conditions are illustrated in Fig 4a. The infiltration happened mainly during the initial 30 min of the process. This was because the level of apoplatic glucose was high (compared with the water film at the leaf surface wherein bacteria were initially present) and a large concentration gradient of glucose caused an enhanced bacterial chemotaxis toward the leaf tissue. During dark conditions, the stomatal opening was not tightly closed as evidenced in Fig. H in S1 Text that shows measured stomatal aperture of spinach leaves at various illumination and dark conditions. This led to an opportunity for the bacteria to infiltrate the leaf in dark condition. Even very narrow openings, slightly larger than a bacterial cell diameter, were shown to be enough for infiltration [21]. However, the amount of infiltration in dark was much less than that in light, as the stomatal size was smaller and photosynthesis was inhibited under dark conditions, leading to a weak chemotaxis.

The distributions of the infiltrated bacteria within the leaf depth are shown in Fig 4b. The infiltration depth for the light condition was more than that in dark. This was because of more nutrient availability at the deeper locations of the leaf tissue when it was exposed to light. Infiltrated bacteria were more concentrated within the stomatal cavity and distributed around as they reached the mesphyll tissue.

The predicted total amounts of infiltrated bacteria into spinach leaves were validated against experimental data obtained in this study (Fig 4c), showing reasonable predictions. The error bar at experimental data shows the standard deviations, and those at predicted data reflect the effect of 30% change in the stomatal size. An increase in the light intensity created more bacterial infiltration into the leaf tissue. This was because the rate of photosynthesis was higher at higher light intensities, which enhanced the bacterial chemotaxis. In this figure, the control condition (Fig. G in S1 Text) confirmed that no natural microbiota at/inside the leaf tissue were able to survive at the LB-agar plates that were supplemented with ampicillin/kanamycin. Therefore, all observed colonies were related to the antibiotic-resistant bacterial strains used in this study.

Light wavelength can also affect the amount of infiltration by altering the amount of nutrients production in the leaf and affecting size of the stomatal aperture. The highest infiltration was observed for the blue light exposure (Fig 4d). A high intensity blue light (similar to that of other lights) can trigger photosynthesis in the guard cells leading to accumulation of sugars and opening of stomata. However, blue light can also serve as a signal in the stomatal opening process: a low intensity blue light is enough to activate the electrogenic H^+^ pumps located at the plasma membrane of the guard cells, leading to membrane hyperpolarization, K^+^ uptake, and stomatal opening [9]. Therefore, the stomatal size under blue light are larger than that of other wavelengths (Fig. H in S1 Text) [22]. Exposure to green light led to the least amount of bacterial infiltration (Fig 4d). This is partly attributed to the size of stomatal opening under green light, which is the least among other light wavelengths (Fig. H in S1 Text) [23]. Also, green light is absorbed less than other wavelengths by chlorophyll pigments within the mesophyll tissue, leading to less photosynthesis inside the leaf and weaker bacterial chemotaxis.

The effect of light intensity and wavelength on the bacterial infiltration might be more complicated than what is considered in the present model. It has been shown that *E. coli* does phototaxis away from blue light [24, 25] and gets more motile at high light intensities [26]. Therefore, a phototactic response and variations in the bacterial motility are also possible to contribute to light-driven bacterial infiltration into the leaf tissue.

#### Effects of motility and chemotaxis on infiltration

Fig 4e compares the total amount of light-driven infiltration for wild-type bacteria (capable to do both chemotaxis and motility) with that of motile-only bacteria. Both experimental and predicted results show that the ability to transport via chemotaxis plays a major role in infiltration. The wild-type *E. coli* K-12 MG1655, capable in both chemotaxis and motility, showed 1.23 log (CFU/g) more infiltration compared with the *CheZ* mutant *E. coli* K-12 BW25113, which was motile-only.

The simulation results showed that chemotaxis toward sugar (glucose) had more than 98% contribution in the total tactic infiltration into the leaf tissue (Fig 4f). This implies that the roles of aerotaxis and chemotaxis toward AI-2 were very insignificant. This is because large concentration gradients of sugar that are developed between the leaf tissue and the water film at the leaf surface cause a large chemotactic flux of bacteria within the stomatal cavity (Fig. P in S1 Text). The *K*_*d*_ values of AI-2 and O_2_ are at least 10 folds smaller than that of glucose (Table A in S1 Text). However, since the concentration gradients of oxygen and AI-2 were small in the system, their respective *K*_*d*_ values (and therefore their respective chemotactic coefficients, *χ*_*cht*_) did not play significant roles in the chemotactic velocity.

#### Effects of leaf side and stomatal defense on infiltration

**Fig 5:**
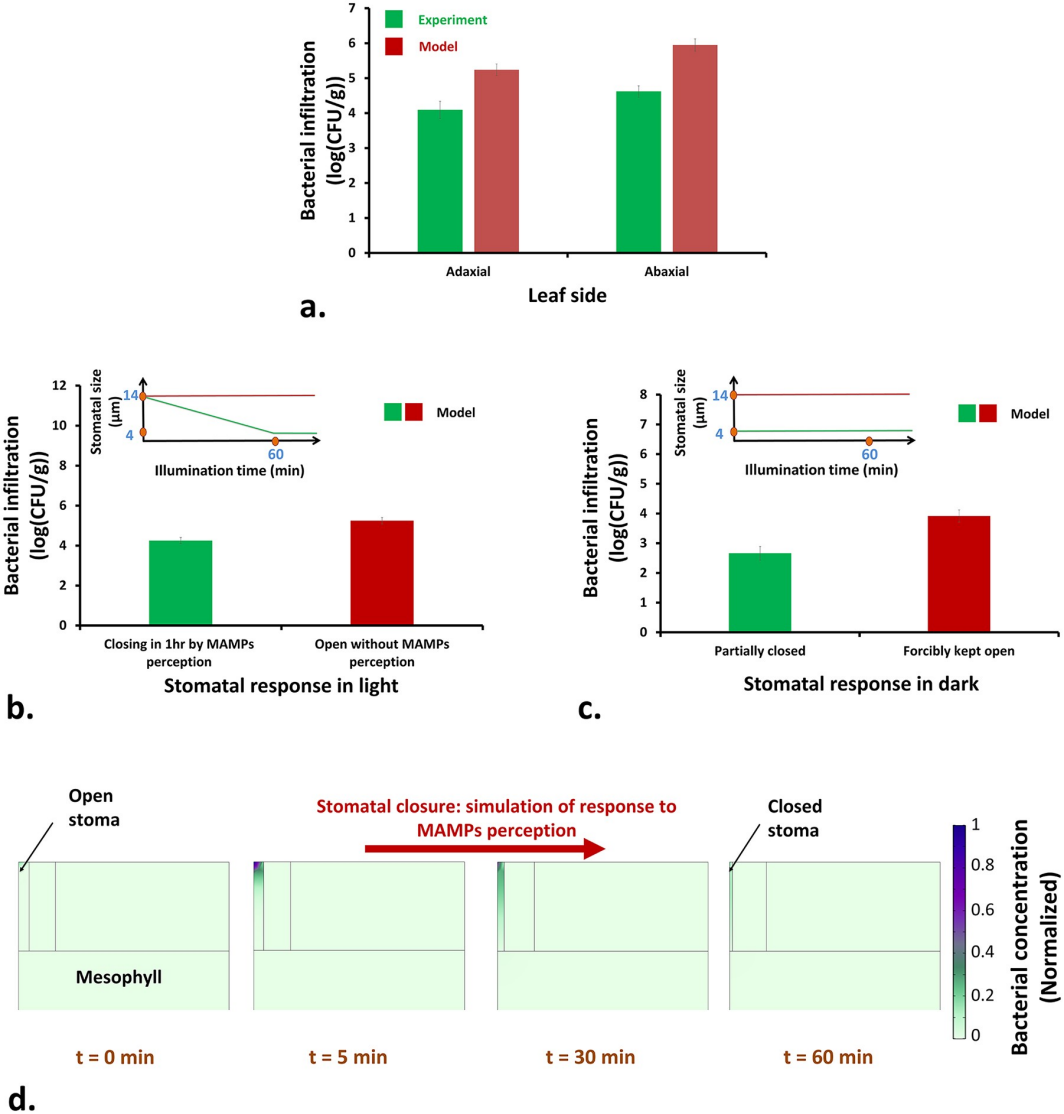
a) Total amounts of bacterial infiltration at white light intensity of 100 *μ*mol/m^2^ · s for two leaf sides. See Fig. J and O (S1 Text) for experimental data presented here. Predicted total amounts of bacterial infiltration for various stomatal responses during b) light and c) dark conditions are shown. The inset graphs show the corresponding variations of the stomatal aperture during illumination time. The experimental data are averages of at least three replications. Error bars on the experimental data represent the standard deviation. For the predicted data, the error bars show the effect of 30% change in the stomatal aperture on the amount of bacterial infiltration. d) Contours of bacterial concentration within the stomatal cavity that is being closed.

Leaf side can play a role in the amount of infiltration. Both experimental and computed results showed an increased bacterial infiltration for the abaxial side of the leaf (Fig 5a). When bacteria infiltrate the abaxial stomata, they face the spongy mesophyll that are not as tightly packed as the palisade tissue. Moreover, the stomatal density at the abaxial side is much higher than that of the adaxial side [27], providing more infiltration routes for bacteria. Therefore, although the photosynthesis is less in spongy regions due to less chloroplast density of the spongy cells (compared to palisade cells), the total amount of infiltration from the abaxial side is higher.

Stomatal behavior depends on a number of biotic and abiotic factors. These external factors can influence the balance of phytohormones such as jasmonic acid (JA), salicylic acid (SA), and abcisic acid (ABA) within the guard cells to affect the work-flow within the stomatal guard cells and regulation of the stomatal opening. In general, dominance of JA signaling over SA and ABA signaling (e.g., in high humidity conditions or under light exposure) favors stomatal opening while a reverse condition can lead to stomatal closure [28]. Plants can sense the bacterial invasion through the pattern-recognition receptors (PRRs) that exist at the extracellular regions of the plant leaf. The PPRs can sense the MAMPs (e.g., components of bacterial surface structures such as flagellin and lipopolysaccharides) and trigger the pathogen-triggered immunity (PTI) that is the first line of the active defense against bacterial invasion [5]. One of the first outputs of the PTI, in response to perception of MAMPs, is stomatal closure. MAMPs perception up-regulates SA signaling and down-regulates JA signaling, leading to stomatal closure against bacterial invasion [28]. Fig 5b shows the amount of bacterial infiltration in light for the situations with or without stomatal defense capability. In the first scenario, the stomatal aperture closes over 1 h (see Fig 5d for visualization). This situation resembles the MAMPs-induced rapid stomatal closure (< 2 h) of various plants in the presence of *E. coli* and *Pseudomonas syringae* pv. tomato [2, 29]. In the second scenario, the stoma remains open in spite of bacterial presence in the medium. This situation is similar to the interaction of *S. enterica* serovar Typhimurium with lettuce leaves for which it was shown that the bacteria did not significantly induce stomatal closure [2]. As is shown in Fig 5b, presence of stomatal defense was effective in decreasing bacterial infiltration into the leaf for about 1-log. However, the stomatal defense is not always successful, since some bacteria such as *P. syringae* are able to override PTI and re-open the closed stomata after a few hours by expression of coronatine (COR), a molecular mimic of jasmonoyl-L-isoleucine (JA-Ile) that mediates stomatal opening [28, 30].

In Fig 5c, the amount of bacterial infiltration for two different stomatal responses in dark condition are illustrated. In the first scenario, stomata are partially closed in the dark, which resembles a natural situation. In the second scenario, the stomata are forcibly kept open in the dark, which is a simulation of applying stomatal opening reagent fusicoccin to the leaf [2, 31], stomatal opening in the dark due to high humidity conditions [30], or when dark-closed stomata are re-opened by COR during *P. syringae* pv. tomato infections [28]. The amount of infiltration in the second scenario was more than 1-log higher than that of the first. The above predictions are in agreement with the experimental findings of Krouptiski et al. [2] who showed that the amount of infiltration of *S. enterica* serovar Typhimurium into iceberg lettuce leaves with forcibly-opened stomata was not significantly high; however, it was higher than that of dark-closed stomata.

### Sensitivity analysis

**Fig 6:**
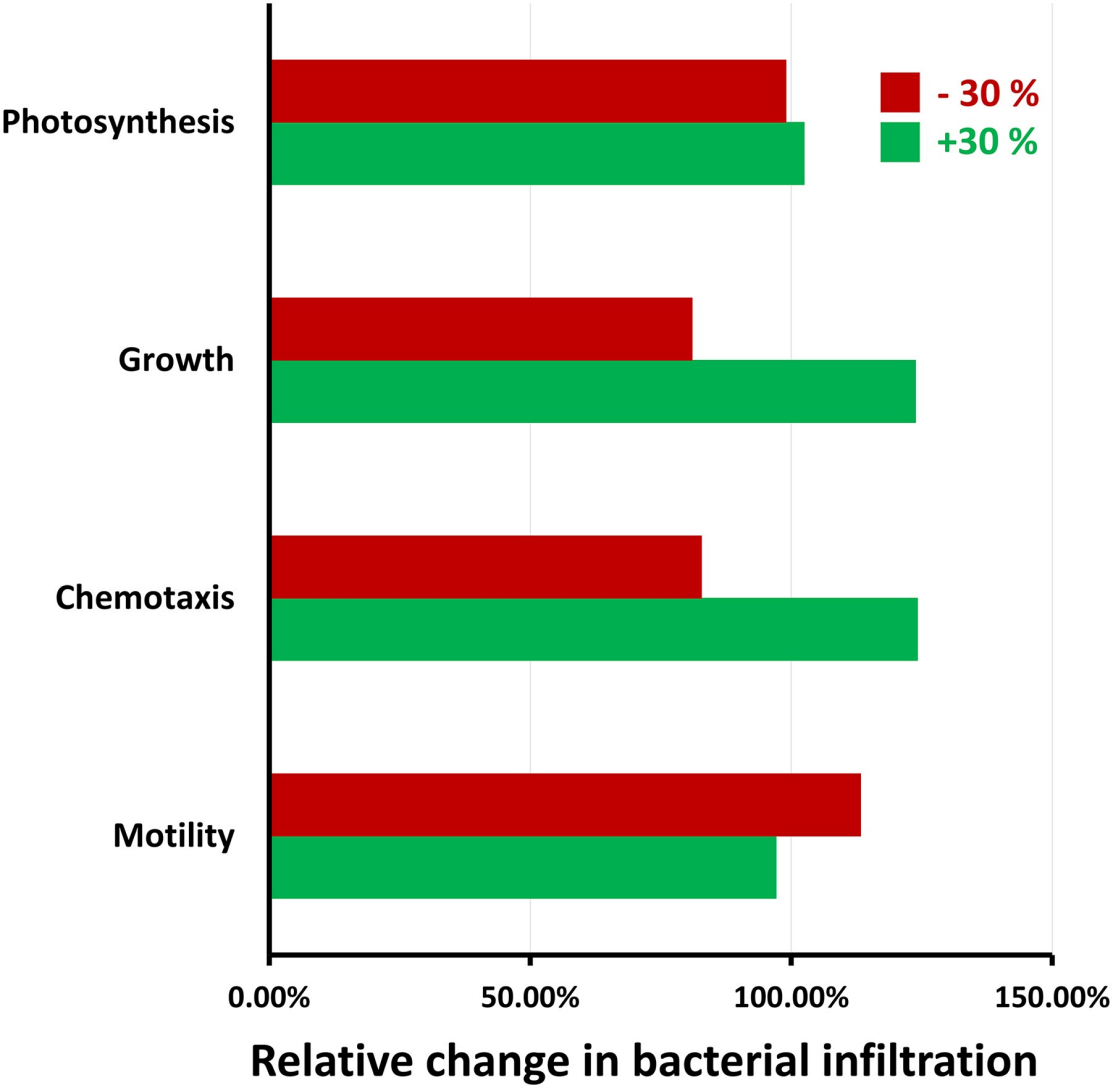
Sensitivity analysis on the leaf and bacterial parameters. Here, the change in the total amount of light-driven bacterial infiltration due to 30% change in the coefficient of random motility, bacterial chemotactic sensitivity coefficient, bacterial growth rate and rate of photosynthesis, was analyzed.

Parametric sensitivity analysis was performed to study the sole effects of motility, chemotaxis, growth, and photosynthesis on the amount of light-driven bacterial infiltration (Fig 6). In this analysis, the total amount of bacterial infiltration due to a certain change in a specific parameter, e.g., coefficient of random motility (while everything else in the model is kept the unchanged), is calculated. Note that the sensitivity analysis was also done on other parameters such as water saturation in the stomatal pore, however the respective results are not reported as they were not significant.

A 30% increase in the coefficient of random motility led to a 3% decrease in infiltration. Motility is a random movement. High motility reduced the chance of bacteria in the leaf surface water film to reach the stomatal pore and infiltrate it. Studies of *E. coli* infiltration through 10-*μ*m diameter capillary arrays (microtubes connecting two chambers containing motility medium) showed that less motile bacteria were better able to enter capillary tubes and pass through them than highly motile bacteria [32], supporting the current predictions.

Increasing the bacterial chemotactic coefficient by 30% created 24% more infiltration. The bacterial chemotactic coefficient is an inherent property of cell type. The above result shows that bacteria with a higher chemotactic coefficient will have a higher chemotactic velocity (Eq 12) toward concentration gradients of photosynthetic products and therefore can infiltrate more. Higher chemotaxis can also happen due to higher concentration gradients of nutrients. Addition of exogenous sugars (thus eliminating the concentration gradients), like glucose and fructose to the bacterial inoculum at the leaf surface, was shown to significantly inhibit bacterial infiltration into iceberg lettuce during illumination [2].

Growth is also a significant factor in increasing the bacterial concentration stemming from infiltration. The effect of growth, of course, depends on the relative time scales of transport and growth, which is described by Damkohler number (Da) [33]. Here, Da is defined as the ratio of the rate of bacterial growth to the rate of bacterial transport. Considering the leaf depth as the characteristic length scale, Da was calculated in the range of 0.01 to 10, where the lower value belongs to a strong chemotactic transport and the higher one belongs to a motile-only transport. This range of Da shows that the growth rate is comparable to the transport rate and thus can affect the bacterial concentration within the leaf tissue.

SWEET transporters limited the rate of sugar transport across the plasma membrane and its availability in the apoplast. Based on model results, a 30% increase in the rate of photosynthesis led to less than 15% increase in the rate of glucose efflux by SWEET transporters, and finally, less than 5% increase in the concentration gradient of sugar. Therefore, the change in the rate of photosynthesis did not significantly impact the amount of infiltration, which implied that the mere existence of photosynthesis, regardless of its rate, is sufficient for promoting the infiltration.

### Big picture: Factors affecting infiltration

**Fig 7:**
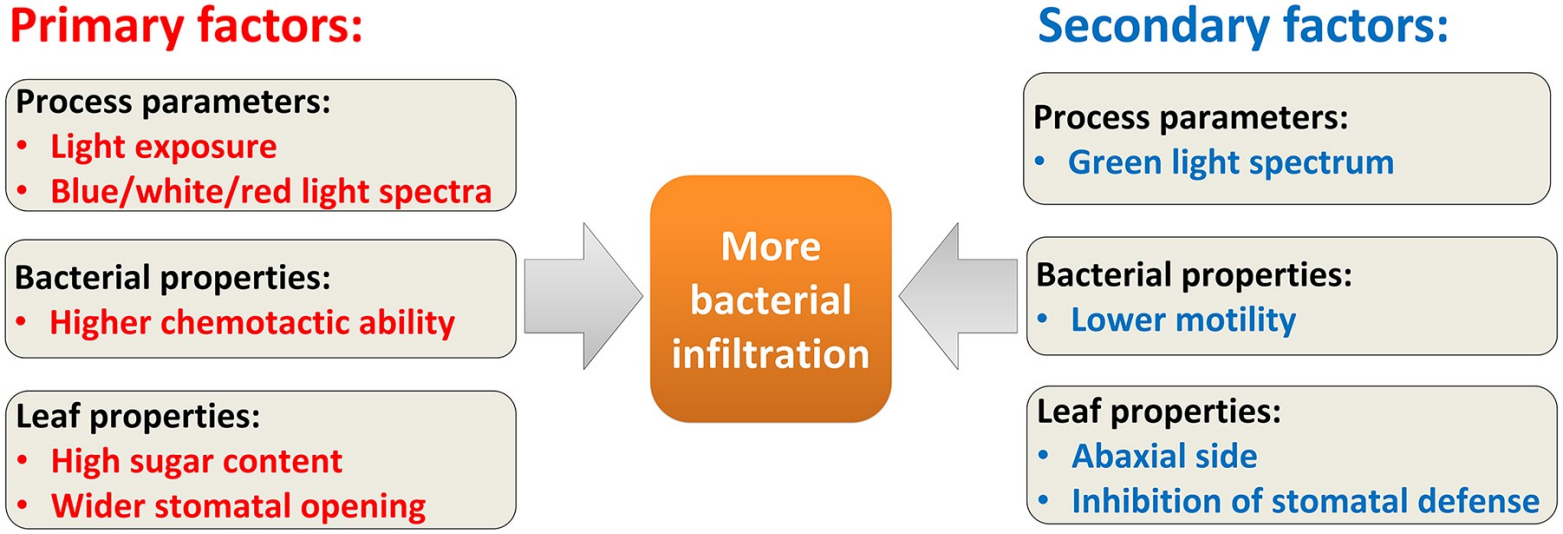
Primary and secondary factors leading to light-driven bacterial infiltration into plant leaves. These factors could be illumination process parameters, leaf properties or bacterial properties. This classification was done qualitatively based on all the computational and experimental findings obtained in this work.

Based on the results presented above (including all computational and experimental data and sensitivity analysis), the primary and secondary factors affecting the amount of infiltration into the leaf tissue are qualitatively chosen and summarized in Fig 7. This figure captures the overall understanding developed using the model and experiments in this study, as well as experimental evidence reported in literature. Primary factors leading to more bacterial infiltration include the presence of blue/white/red light and photosynthesis, higher initial sugar content due to pre-exposure to light, high chemotactic ability of bacteria, and wider stomatal size. The secondary factors include presence of green light exposure, leaf abaxial side, lower bacterial motility, and inhibition of stomatal defense.

### Conclusions

A mechanistic model of light-driven infiltration of bacteria into stomatal openings of plant leaves was presented. The concentration of photosynthetic sugar (glucose/sucrose) in the apoplast of the leaf tissue was predicted reasonably accurately, as compared with the experimental literature data. Based on the model results and experimental findings, it was shown that presence of light with moderate intensity (100 *μ*mol/m^2^ · s) is sufficient to induce chemotactic invasion of bacteria toward photosynthetic products within the leaf tissue. Bacteria were able to infiltrate the leaf tissue during dark conditions. However, the amount of infiltration during dark condition was significantly less than that in the light. Blue light induced the highest amount of infiltration, while the green light caused the lowest. The ability of bacteria to transport via taxis was a major factor in infiltration. Chemotaxis toward glucose was much more significant than aerotaxis or chemotaxis toward AI-2. Higher motility caused a decrease in infiltration as it decreased the chance of bacteria in the leaf surface water film to reach the stomatal pore. Plant stomatal defense, induced by perception of MAMPs, was shown to play a role in inhibition of bacterial invasions into the leaf apoplast.

## Materials and methods

In this section, after an overview of the computational solution domain and assumptions, the physics-based model for light-driven chemotactic infiltration of bacteria into the leaf tissue will be elaborated. This is followed by the details of the microbiological experiments and microscopy imaging approach used for validation and support of the model predictions.

### Computational schematic and assumptions

A 2D-axisymmetric domain around one stomatal pore, including the leaf tissue and a water film at the leaf surface, was considered as the solution domain (Fig 2a and 2b). Transport and interactions of seven species including CO_2_, O_2_, ${\text{HCO}}_{3}^{-}$, symplastic glucose/sucrose, apoplastic glucose/sucrose, bacteria, and AI-2 were studied in an isothermal condition. For all species other than bacteria, a diffusion-reaction equation described their time and spatial evaluations. For bacteria, in addition to a diffusion transport term that described their motile (diffusion-like) motion, a convective term defined their tactic (convective-like) motion in the free water phase. The leaf tissue was assumed to be a smeared porous medium containing solid, gas and water phases. In the porous media formulation, each phase was known by its volume fraction. Due to uncertainty about the situation of gas and free water within the stomatal cavity, this region was also considered as a porous zone and each phase inside that was determined by its own volume fraction. The leaf surfaces were assumed to be impermeable to mass transfer due to the presence of the waxy cuticle layer. Chloroplasts and mitochondria were known by their respective volume fractions (which depended on the plant cell type and location) within leaf tissue. Within guard cell and mesophyll regions, there were photosynthesis, photorespiration, respiration, and CO_2_ hydration reactions. For epidermis layers, only respiration and CO_2_ hydration reactions were considered. A reaction term described the exchange of sugar between symplast and apoplast through SWEET transporter proteins. Bacterial uptake of apoplastic glucose and O_2_ were also modeled as a reaction term. Movements of stomatal aperture (to simulate stomatal response to light exposure or MAMPs perception) was performed by using a moving mesh approach in which the velocity of the stomatal opening/closure is prescribed at the aperture boundary. The free movement of the mesh inside the domain (see Fig 2b for the moving mesh region) was obtained using the Laplace smoothing method.

### Governing equations

An overview of the model, including all involved species and their interconnections are shown in Fig. C in S1 Text. Temporal and spatial evolution of each species was described by a diffusion-reaction equation. For bacteria, a convective term was also added to the transport equation to account for the chemotactic motion toward photosynthetic products.

#### Basic definitions

The solution domain included a leaf section and a water film at the leaf surface (Fig 2b). Within the leaf section, a total porosity, *ϕ*, was defined as the volume fraction occupied by all fluid phases:

$$\phi =\frac{1}{\delta V}\sum_{i}^{}\delta V_{i}$$ (1)

Here, *i* = *wf*, *wb*, *g* represents free (intercellular) water, bound (intracellular) water, and gas phases, respectively, and *δV*_*i*_ is the volume occupied by the *i*th phase within a representative elementary volume (REV), *δV*. Each fluid has a saturation that is defined as a fraction of the total fluid volume within the REV:

$$S_{i}=\frac{\delta V_{i}}{\phi \;\delta V}$$ (2)

Therefore, *S*_*g*_ + *S*_*wf*_ + *S*_*wb*_ = 1. Volume fractions of *j*th cell organelles (i.e., chloroplasts and mitochondria) are defined by a sub-saturation coefficient as a fraction of the bound water volume:

$$\gamma_{j}=\frac{\delta V_{j}}{S_{wb}\phi \;\delta V}$$ (3)

Concentration of species (CFU/m^3^ for bacteria and mol/m^3^ for others), *i*, within the computational domain is defined as:

$$c_{i}=S_{g}\phi c_{i,g}+S_{wf}\phi c_{i,wf}+S_{wb}\phi c_{i,wb}$$ (4)

where *c*_*i*,*g*_, *c*_*i*,*wf*_ and *c*_*i*,*wb*_ are concentrations in the gas, free water and bound water phases, respectively. For some species, one or two of these concentrations can be zero. For instance, for bacteria, *c*_*i*,*g*_ = 0 and *c*_*i*,*bw*_ = 0. Solution of a transport equation for species *i* gives the distribution of *c*_*i*_ within a REV. Then Eq 4 can be used to solve for the concentration values in each phase.

#### Transport of CO_2_

As light drives the stomata to open, disolved CO_2_ in the water film at the leaf surface can diffuse into the porous leaf tissue (Fig 1) and be consumed in the chloroplasts during photosynthesis. Also, due to photorespiration in chloroplast and dark respiration in the mitochondria, some of the photosynthetic products will be oxidized, producing CO_2_. In addition, CO_2_ may be hydrated in the aqueous medium. Assuming CO_2_ in the gas and liquid phases are in equilibrium [34], the transport equation for CO_2_ in the solution domain can be written as (see S1 Text for derivation):

$$\frac{\partial c_{{co}_{2}}}{\partial t}=\nabla \cdot \left(D_{{co}_{2},eff}\nabla c_{{co}_{2}}\right)-R_{phs}\gamma_{chl}S_{wb}\phi +R_{res}\gamma_{mit}S_{wb}\phi -R_{hyd}\left(S_{wf}+S_{wb}\right)\phi$$ (5)

where *R*_*phs*_ is the net photosynthesis rate, i.e., CO_2_ fixation rate in the chloroplasts (mol/m^3^ · s) (Eq 15), *R*_*res*_ is the rate of dark respiration in mitochondria (mol/m^3^ · s) (Eq 19), *R*_*hyd*_ is the rate of CO_2_ hydration in water phase (mol/m^3^ · s) (Eq 14), and *D*_*co*2,*eff*_ is the effective diffusivity of CO_2_ in the leaf (m^2^/s) that is defined in Eq. S7 (S1 Text). Note that in the water film at the leaf surface, *R*_*phs*_ and *R*_*res*_ are zero, and *D*_*co*2,*eff*_ is equal to the molecular diffusivity of CO_2_ in water.

#### Transport of bicarbonate

Bicarbonate (${\text{HCO}}_{3}^{-}$) diffuses within the water phase. It is generated from hydration of CO_2_ and makes it unavailable for photosynthesis in chloroplast. Transport equation for ${\text{HCO}}_{3}^{-}$ is:

$$\frac{\partial c_{{HCO}_{3}^{-}}}{\partial t}=\nabla \cdot \left(D_{{HCO}_{3}^{-},w}\nabla c_{{HCO}_{3}^{-}}\right)+R_{hyd}\left(S_{wf}+S_{wb}\right)\phi$$ (6)

where *R*_*hyd*_ is the rate of hydration of CO_2_ (mol/m^3^ · s) (Eq 14).

#### Transport of O_2_

Oxygen is absorbed by the water phase from intercellular gas and within cells, it is consumed during respiration in mitochondria and generated during photosynthesis. Bacteria can consume oxygen as a nutrient. Assuming O_2_ in the gas and liquid phases are in equilibrium [34], the transport of O_2_ in the solution domain is written as (see S1 Text for derivation):

$$\frac{\partial c_{o_{2}}}{\partial t}=\nabla \cdot \left(D_{o_{2},eff}\nabla c_{o_{2}}\right)+R_{phs}\gamma_{chl}S_{wb}\phi -R_{res}\gamma_{mit}S_{wb}\phi -R_{o_{2},bac}S_{wf}\phi$$ (7)

where *D*_*o*2,*eff*_ is the effective diffusivity of O_2_ in the leaf (m^2^/s) that is defined by Eq. S7 (S1 Text) and *R*_*o*2,*bac*_ is the rate of uptake of O_2_ by bacteria (mol/m^3^ · s) (Eq 21). Note that in the water film at the leaf surface, *R*_*phs*_ and *R*_*res*_ are zero, and *D*_*o*2,*eff*_ is equal to the molecular diffusivity of O_2_ in water.

#### Transport of symplastic sugar

Light exposure triggers sugar synthesis in the plant cells consisting of chloroplasts. Glucose/sucrose transports through plasmodesmata and membrane proteins (i.e., SWEET transporters) [10, 35] to be available in the apoplast. Transport of symplastic sugar is modeled as:

$$\frac{\partial c_{s,sug}}{\partial t}=\nabla \cdot \left(D_{sug,w}\nabla c_{s,sug}\right)+\alpha R_{phs}\gamma_{chl}S_{wb}\phi -R_{res}\gamma_{mit}S_{wb}\phi -R_{SWEET}S_{wb}\phi$$ (8)

where subscript *s, sug* denotes symplastic glucose or sucrose, and *R*_*SWEET*_ is the rate of sugar transport across plasma membrane through SWEET transporters (mol/m^3^ · s) (Eq 20). To generate one molecule of glucose and sucrose, 6 and 12 molecules of CO_2_ are consumed, respectively; therefore, $\alpha_{gluc}=\frac{1}{6}$ and $\alpha_{suc}=\frac{1}{12}$. Note that sucrose transport was studied here only to prove the validity of this framework in predicting sugar synthesis.

#### Transport of apoplastic sugar

Once the synthesized sugar reaches apoplast, it is available to the bacteria that have infiltrated the leaf tissue. Transport of apoplastic glucose/sucrose is modeled as:

$$\frac{\partial c_{a,sug}}{\partial t}=\nabla \cdot \left(D_{sug,w}\nabla c_{a,sug}\right)+R_{SWEET}S_{wf}\phi -R_{sug,bac}S_{wf}\phi$$ (9)

where subscript *a, sug* denotes apoplastic glucose or sucrose, and *R*_*sug*,*bac*_ is the rate of apoplastic sugar uptake by bacteria (mol/m^3^ · s) (Eq 21). Note that sucrose transport was studied here only to prove the validity of this framework in predicting sugar synthesis. For sucrose, the last term on the right-hand side of Eq 9 is zero, since many microorganisms, including *E. coli*, mainly uptake glucose as their primary carbon source (see S1 Text for more details).

#### Transport of AI-2

AI-2 is a chemoattractant molecule that is secreted by *E. coli* (Fig. Bc in S1 Text) and enhances chemotaxis toward exogenous nutrients like glucose [36]. The transport of AI-2 in the leaf tissue is given by:

$$\frac{\partial c_{AI2}}{\partial t}=\nabla \cdot \left(D_{AI2,w}\nabla c_{AI2}\right)+R_{AI2}S_{wf}\phi$$ (10)

where *R*_*AI2*_ is net rate of AI-2 production by bacteria (mol/m^3^ · s) (Eq 22).

#### Transport of bacteria

The continuum Keller-Segel model [6] is used to describe the distribution of bacteria. Within the leaf, bacteria can only transport in the free water layer at the surface of the mesophyll cells (Fig 1c). The mechanisms of bacterial transport include a random diffusion-like motion (motility) as well as chemotactic transport within free water phase. The mass balance for bacteria is:

$$\frac{\partial c_{bac}}{\partial t}+\nabla \cdot (c_{bac}\sum_{i}u_{cht,i})=\nabla \cdot \left(\eta_{bac}\nabla c_{bac}\right)+R_{bac,gr}S_{wf}\phi$$ (11)

where *i* represents either nutrients (i.e., glucose and O_2_) and chemoattractants secreted by bacteria (i.e., AI-2), *c*_*bac*_ is the concentration of bacteria (cell/m^3^ or CFU/m^3^), *η*_*bac*_ is the coefficient of random motility (i.e., bacterial diffusion coefficient) (m^2^/s), and *R*_*bac,gr*_ is the rate of bacterial growth (CFU/m^3^ · s) (Eq 23). The rate of bacterial death is ignored here as the bacteria are assumed to be in their exponential phase of their growth.

The chemotaxtic velocity of bacteria is in the direction of the concentration gradients of species *i*, including glucose, O_2_ and AI-2:

$$u_{cht,i}=\chi_{cht,i}\frac{\nabla c_{i}}{c_{i}}(1-\frac{c_{bac,wf}}{c_{bac,wf,max}})$$ (12)

At high concentrations of nutrients or chemoattractants, bacteria sense the absolute gradients (∇*c*_*i*_), while at low concentrations, they sense the logarithmic gradients (∇ log*c*_*i*_ = ∇*c*_*i*_/*c*_*i*_). Following Curk et al. [37], a threshold value of 0.01 mol/m^3^ was adopted to switch between these two modes of gradient sensing. At high concentrations, bacterial swimming path becomes limited. The inhibition function in Eq 12 presents a simple approach to account for the effect of bacterial concentration on bacterial chemotactic velocity. The maximum bacterial concentration, *c*_*bac*,*wf*,*max*_, was adopted to be 1 × 10^18^ CFU/m^3^ [38]. The bacterial chemotactic coefficient (m^2^/s), *χ*_*cht*_, is defined as [6]:

$$\chi_{cht,i}=\chi_{0}\frac{K_{d,i}}{{\left(K_{d,i}+c_{i}\right)}^{2}}$$ (13)

where *χ*_*0*_ is chemotactic sensitivity coefficient (m^2^/s), and *K*_*d*_ is the receptor-ligand binding dissociation constant (mol/m^3^). Estimations of numerical values of these parameters for *E. coli* can be found in Tindall et al. [6], Ford and Lauffenburger [39], Ford et al. [40], Jani et al. [41] and Delgado-Nixon et al. [42]. The porous structure of the leaf can affect bacterial migration and confine their motile and tactic movements [43]. To include this confinement effect, the amounts of coefficient of random motility, *η*_*bac*_, and chemotactic sensitivity coefficient, *χ*_*0*_, in the porous media were assumed to be two orders of magnitude less than that in the water layer.

#### Rate of hydration of CO_2_

Carbonic anhydrase (CA) can catalyze the hydration of CO_2_ to ${\text{HCO}}_{3}^{-}$. However, its effect on the rate of photosynthesis was not significant [13]. In the absence of carbonic anhydrase, the rate of hydration of CO_2_ is defined as:

$$R_{hyd}=k_{1}c_{{co}_{2},w}-k_{2}\frac{c_{H^{+}}c_{{HCO}_{3}^{-},w}}{K_{hyd}}$$ (14)

where *k*_*1*_, *k*_*2*_ and *K*_*hyd*_ are rate constants of the reaction. The concentration of $c_{H^{+}}$ in (mol/m^3^) was estimated based on the values of pH within the leaf and water layer, i.e., $\text{pH}=-{\text{log}}_{10}\left(10^{-3}\;c_{H^{+}}\right)$.

#### Rate of photosynthesis

Photosynthesis is one of the most studied and best understood physiological processes [44]. Notable details of the photosynthesis process and machinery are very briefly discussed in S1 Text, and illustrated in Fig. Ac in S1 Text. So far, very detailed biochemical models, including light reactions, proton transport, enzymatic reactions and regulatory functions, have been developed [45, 46]. However, due to a high level of complexity, these models cannot be used to predict photosynthesis in leaf-level applications [44]. Instead, the biochemical model of Farquhar et al. [8] (i.e., the FvCB model) can be effective for this purpose. The art of this model is that it makes no attempt to model all processes of photosynthesis but rather focuses on a few key processes involved in C3 photosynthesis [44]. Considering FvCB model, the net photosynthesis rate (mol/m^3^ · s), *R*_*phs*_, in the chloroplasts of the C3 plant cells is described as:

$$R_{phs}=\text{min}\left\{A_{c},A_{j},A_{p}\right\}$$ (15)

$$A_{c}=\frac{\left(p_{{co}_{2}}-\Gamma^{*}\right)V_{c,max}}{p_{{co}_{2}}+K_{m,{co}_{2}}\left(1+p_{o_{2}}/K_{m,o_{2}}\right)}$$ (16)

$$A_{j}=\frac{(p_{{co}_{2}}-\Gamma^{*})J}{4p_{{co}_{2}}+8\Gamma^{*}}$$ (17)

$$A_{p}=\frac{3T_{p}^{*}}{1-\Gamma^{*}/p_{{co}_{2}}}$$ (18)

where *A*_*c*_ is the RuBisCO-limited rate of CO_2_ assimilation (mol/m^3^ · s), *A*_*j*_ is the electron transport-limited rate of CO_2_ assimilation (mol/m^3^ · s), and *A*_*p*_ is triose phosphate utilization-limited (TPU-limited) rate of CO_2_ assimilation (mol/m^3^ · s). In Eq 16, $p_{{co}_{2}}$ and $p_{o_{2}}$ are CO_2_ and O_2_ partial pressures (Pa) in chloroplast, *V*_*c*,*max*_ is the maximum carboxilation capacity of RuBisCO (mol/m^3^ · s), $K_{m,co_{2}}$ and $K_{m,o_{2}}$ are Michaelis-Menten constants of RuBisCO for CO_2_ (during photosynthesis) and O_2_ (during photorespiration) (Pa), and Γ* is CO_2_ compensation point without dark respiration (Pa). In Eq 17, *J* is the volumetric rate of electron transport (mol/m^3^ · s) that includes the effects of light intensity and wavelength on the rate of photosynthesis. In Eq 18, $T_{p}^{*}$ is the volumetric TPU rate (mol/m^3^ · s). Details of the constitutive equations used to calculate *A*_*c*_, *A*_*j*_, and *A*_*p*_ are discussed in S1 Text.

#### Rate of respiration

Based on the available values for the dark respiration at 25°C [8, 13], the following equation was used to describe temperature dependence of the dark respiration (mol/m^3^ · s), *R*_*res*_:

$$R_{res}=1.1\times 10^{-6}\text{exp}(\frac{66405(T-298)}{298RT})\alpha_{t}$$ (19)

To convert the rate of respiration to a volumetric value, *α*_*t*_ was assumed to be the reciprocal of the leaf thickness (1/m).

#### Rate of sugar efflux by SWEET transporters

SWEET proteins are energy-independent transporters [10, 35, 47] and transport of glucose/sucrose across them is facilitated by molecular diffusion. The rate of sugar efflux by SWEET transporters depends on the concentration gradient across plasma membrane, population density of the transporters on the plasma membrane, and level of saturation of the transporter. The volumetric rate of sugar transport (mol/m^3^ · s) across plasma membrane through SWEET transporters can be written as:

$$R_{SWEET}=P_{sug}(c_{s,sug}-c_{a,sug})A_{SWEET}\rho_{SWEET}(\frac{c_{s,sug}}{K_{SWEET}+c_{s,sug}})\alpha_{p}$$ (20)

The permeability (m/s) of a SWEET transporter to the sugar of interest was estimated from diffusion coefficient of the sugar in water phase and the thickness of the plasma membrane (7 nm) [48] as *P*_*sug*_ = *D*_*sug*,*w*_/*l*_*plm*_. In the above equation, *A*_*SWEET*_ is the pore surface area of the SWEET transporter (m^2^), *ρ*_*SWEET*_ is the population density of SWEET transporters at the plasma membrane of the plant cells (transporter/m^2^), *K*_*SWEET*_ is the half-saturation constant for the transporter (mol/m^3^), and *α*_*p*_ is the specific surface area (m^2^/m^3^) of the porous structure of the leaf.

#### Rate of uptake of glucose and oxygen by bacteria

The rate of uptake of glucose and O_2_ by bacteria can be modeled as [6]:

$$R_{i,bac}=\zeta_{gr,i}c_{bac}Y_{i/bac}f_{QS}$$ (21)

where *i* denotes apoplastic glucose or O_2_, *ζ*_*gr*,*i*_ is the bacterial growth rate (1/s) defined in Eq 24, *Y*_*i*/*bac*_ is the yield coefficient of nutrients on bacteria (mol/cell), and *f*_*QS*_ is a switch function that represents the effect of quorum sensing of signaling molecules (e.g., indole) during biofilm formation. It can be defined as a Hill function [49] to show state transition in bacterial biofilms. However, as the duration of the process of interest here (2 h) is much shorter than the time-scale for bacteria to reach stationary phase [50] and develop biofilms (≫ 2 h), one can write *f*_*QS*_ = 1. Details of pathways underlying glucose uptake by bacteria are briefly discussed in S1 Text and illustrated in Fig. Bd in S1 Text.

#### Rate of synthesis of AI-2

Synthesis of AI-2 by *E. coli* increases during exponential phase of the cells’ growth. However, as cells reach the stationary phase, they uptake the extracellular AI-2 [51, 52] (Fig. Bc in S1 Text). When glucose is present in the growth medium, synthesis of AI-2 in the exponential phase is boosted, while its uptake in the stationary phase is weakened [53]. Since in this study bacteria are always in their exponential phase, only the synthesis of AI-2 is modeled and its uptake by bacteria is ignored:

$$R_{AI2}=k_{1,AI2}c_{bac}$$ (22)

Here, *R*_*AI*2_ is the rate of synthesis of AI-2 by bacteria (mol/m^3^ · s). The *k*_*1*,*AI2*_ is AI-2 synthesis rate constant (moll/cell · s) whose value depends on the presence of glucose in the medium. The estimated values for this rate constant based on the experimental and simulation data of Xu et al. [54], Wang et al. [51] and Li et al. [53] are included in Table A (S1 Text).

#### Rate of bacterial growth

The rate of bacterial growth as a result of nutrients uptake is:

$$R_{bac,gr}=\zeta_{gr}c_{bac}f_{QS}$$ (23)

The bacterial growth rate constant (1/s), *ζ*_*gr*_, is defined using Monod kinetics [7]:

$$\zeta_{gr}=\zeta_{max,gr}\prod_{i}^{}\frac{c_{i}}{K_{s,i}+c_{i}}$$ (24)

Here, *i* stands for glucose and O_2_, *ζ*_*max*,*gr*_ is the maximum growth rate constant (1/s), and *K*_*s,i*_ is the Monod half saturation constant (mol/m^3^). Note that here *f*_*QS*_ = 1, as bacteria are in their exponential phase of growth.

### Boundary and initial conditions

Table A (S1 Text) shows all numerical values used for boundary and initial conditions in this study. Initial concentrations of CO_2_ and O_2_ in the solution domain were calculated based on the saturation of each phase and Henry’s law. Their concentrations in the gas phase were equal to atmospheric levels. Initial concentrations of sugars in spinach leaves were obtained from Voitsekhovskaja et al. [18]. For ${\text{HCO}}_{3}^{-}$, initial concentration in the water phase was set as 0.001 mol/m^3^ (assumed from Allakhverdiev et al. [55]). Bacterial concentration was initially set to 1 in the water film at the leaf surface (by normalizing to the bacterial population in the inoculum reported in the Experimental procedure section) and was set to 0 in the leaf tissue. Concentration of AI-2 was initially zero in the entire domain.

An overview of the boundary conditions is shown in Fig 2b. The constant concentration boundary condition at the top boundary of the water film is defined as:

$$c_{i}=c_{i,\infty }$$ (25)

where *i* stands for CO_2_, O_2_, and bacteria. Note that for CO_2_ and O_2_, the concentrations in the water phase can be estimated by Henry’s law based on their partial pressures in the gas phase [13]:

$$c_{co_{2},\infty }=RTK_{H,co_{2}}c_{co_{2},g}$$ (26)

$$c_{o_{2},\infty }=RTK_{H,o_{2}}c_{o_{2},g}$$ (27)

where $K_{H,co_{2}}$ and $K_{H,o_{2}}$ are the Henry’s law constants for CO_2_ and O_2_ (mol/m^3^ · Pa), respectively, *R* is the universal gas constant (J/mol · K), and *T* is temperature (K). The no flux condition for any species, *i*, applied to several boundaries in the solution domain (see Fig 2b) is written as:

$$-D_{i,f}\frac{\partial c_{i}}{\partial n}=0$$ (28)

where subscript *f* stands for fluid phase and *n* denotes the normal direction to the boundary.

### Input parameters

Input data for the simulations are shown in Table A (S1 Text). Details of the input parameters for the FvCB model of photosynthesis (Eqs 15 to 18) are discussed in S1 Text.

### Solution procedure

The governing equations were solved using a commercial finite element package, COMSOL Multiphysics version 5.4 (COMSOL Multiphysics Burlington, MA). The maximum time-step size was varied between 0.001 s to 0.1 s. The relative and absolute tolerances were 0.001 for all computations. A mesh of 2477 triangular elements was used for the 2D axisymmetric model for which the maximum element size was 8 *μ*m within the mesophyll tissue and far from the stomatal cavity. MUMPS direct solver was used to solve the algebraic equations resulting from the finite element method. Run time for the simulations ranged from a few to several minutes on a Windows machine with 32 GB of RAM and 2 GHz dual core Intel Xeon CPU E5-2620 processor.

### Experimental procedure

In order to validate the model predictions for the amount of bacterial infiltration, microbiological experiments were performed using baby spinach leaves. A brief description of the experimental procedure is described here and more details are available in S1 Text. The most important experimental steps to determine the total amount of infiltrated bacteria into leaves were (Fig. E in S1 Text): 1) inoculum preparation, in which a cell suspension containing *E. coli* (K-12 MG1655 or BW25113 (*Δ CheZ*)) with a population of ∼ 10^8^ CFU/ml was prepared, 2) leaf surface inoculation, in which the leaf surfaces were spot inoculated with the bacterial suspension to reach an initial population of ∼ 3 × 10^7^ CFU/g, 3) leaf illumination, in which the leaf surfaces were exposed to white/blue/red/green light with intensity of 100 *μ*mol/m^2^ · s, or kept in the dark, 4) leaf surface sterilization, in which the leaf surfaces were washed by sterile 0.85% NaCl (saline) solution and 70% ethanol multiple times to remove any surface bacteria, 5) sample crushing, in which the surface-sterilized leaves were crushed in a sterile bowl, 6) crushed leaf homogenization in 0.85% NaCl (saline) solution, 7) serial dilution, 8) plating of the diluted extract, and 9) plates incubation at 30°C for 24 h. In addition to the microbiological experiments, stomatal aperture of spinach leaves under exposure of different illumination conditions were examined using microscopy imaging (S1 Text).

## Supporting information

S1 Text The supporting information includes an overview of the most relevant biological aspects related to leaf and bacteria, an overview of the model, derivation of transport equations for CO_2_ and O_2_, and input parameters of the model. Also, it includes information about bacterial strains and procedure for inoculum preparation, leaf inoculation and light exposure, bacterial infiltration assay, microscopy imaging of stomatal aperture, evidence of experimental quantification of bacterial infiltration into spinach leaves, and model predictions for bacterial flux inside stomatal cavity.
(PDF)
