# Supplementary material for: Mechanistic modeling of light-induced chemotactic infiltration of bacteria into leaf stomata
Source: PLoS Comput Biol. 2020 May 8;16(5):e1007841. doi: 10.1371/journal.pcbi.1007841 (PMC7209104; doi:10.1371/journal.pcbi.1007841)
Supplement: S1 Text — Also, it includes information about bacterial strains and procedure for inoculum preparation, leaf inoculation and light exposure, bacterial infiltration assay, microscopy imaging of stomatal aperture, evidence of experimental quantification of bacterial infiltration into spinach leaves, and model predictions for bacterial flux inside stomatal cavity. (PDF) [file pcbi.1007841.s001.pdf]

Mechanistic modeling of light-induced chemotactic infiltration of bacteria into leaf stomata (Supporting Information)

Mohsen Ranjbaran[1], Mina Solhtalab[1,2], Ashim K. Datta[1,*]
[*]*Email of the corresponding author:* akd1@cornell.edu

[1]*Department of Biological and Environmental Engineering, College of Agriculture and Life Sciences, Cornell University, Ithaca, NY 14853, USA*

[2]*Department of Civil and Environmental Engineering, McCormick School of Engineering and Applied Science, Northwestern University, Evanston, Il 60208, USA*

April 15, 2020

Biological aspects

The model includes various biological factors related to plant and bacteria, and they are briefly discussed here. These details are only to provide an idea about the underlying subcellular events that have been included in the modeling framework presented in this manuscript.

Leaf

A typical leaf section is shown in Fig. Aa. It includes a mesophyll tissue in the middle and two epidermis layers at top and bottom. The leaf surfaces are covered by a waxy cuticle. Stomatal openings are responsible for selective mass exchange between the leaf tissue and outside environment based on various biotic and abiotic environmental conditions [2-4]. Inside plant cells (Fig. Ab), the cytoplasm is covered by a plasma membrane and a cell wall. The majority of the cytoplasm volume is occupied by vacuoles that mainly contain water. Guard cells and mesophyll cells contain chloroplasts that are responsible for photosynthesis (production of organic nutrients). All cells within the leaf section have mitochondria that are responsible for respiration (production of energy from oxidation of organic nutrients). Water and nutrients can move from one cell to another through plasmodesmata, known as symplastic transport. In contrast, mainly in apoplastic phloem loaders such as spinach leaves, nutrients are also available in the apoplastic region [5]. Here, any water outside of the plasma membrane is called free water whereas intracellular water is called bound water. During photosynthesis (Fig. Ac), light reactions occur within thylakoid membranes in which water is consumed as an electron donor and releases oxygen, hydrogen ions and electrons. The electrons are supplied to two successive electron transport chains that end up with production of ATP and NADPH to contribute in carbon fixation. Calvin cycle uses these molecules to convert carbon dioxide (CO_2) to triose phosphates (TPs) (*i.e.*, three-carbon sugar-phosphate molecule) that are building blocks for sugar production. The enzyme that catalyzes this carbon fixation step is RuBP carboxilase/oxygenase, or RuBisCO. The cycle runs three times to generate one molecule of triose-phosphate that is then provided to the plant to synthesize starch and sugars (e.g., sucrose).

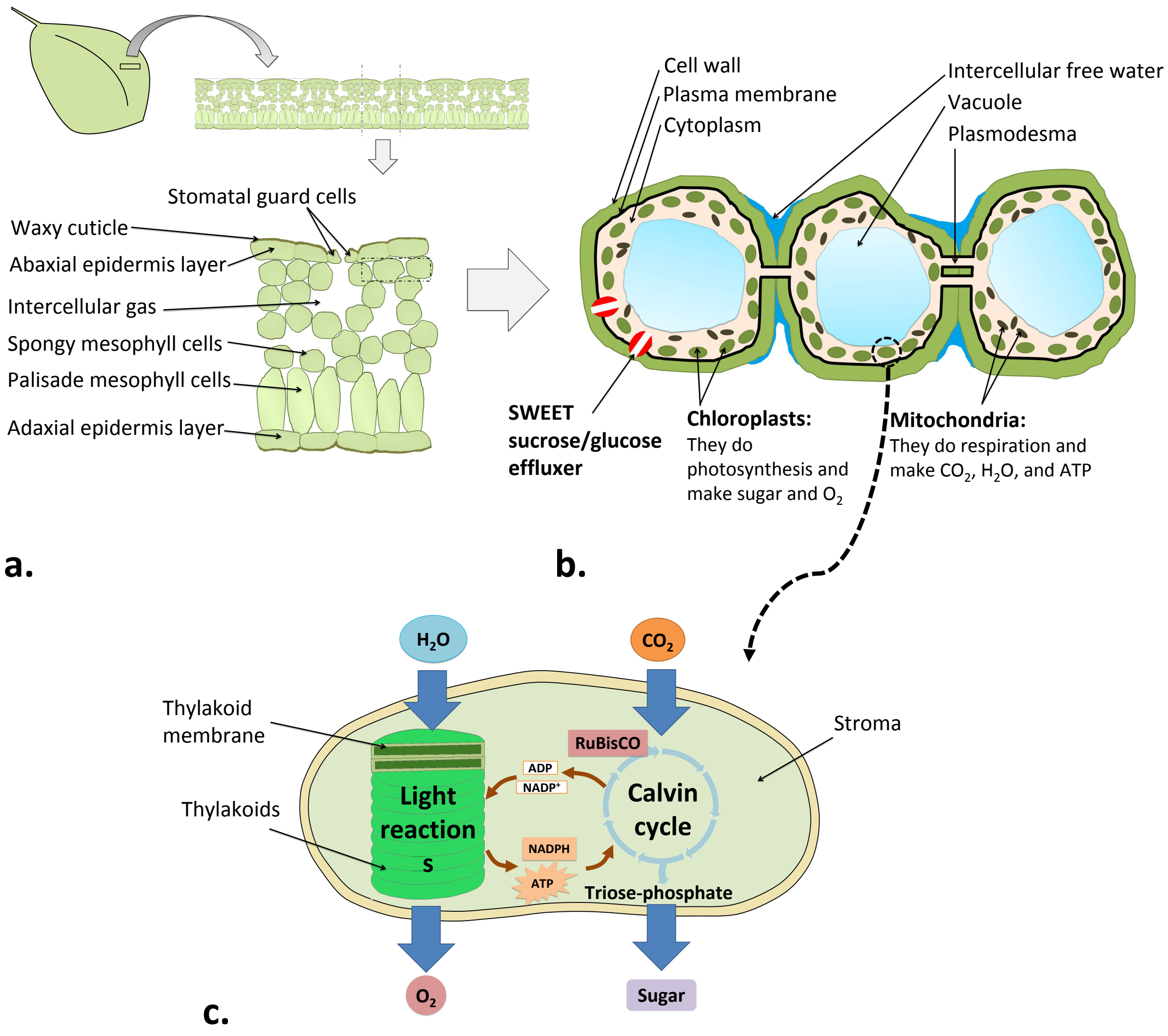

Figure A: a) A leaf cross section with b) an illustration of cell organs. c) A schematic of a chloroplast with various functions that lead to photosynthesis in plant cells. The chloroplast image inspired by Campbell et al. [1]. Note that only organs and functions that are of interest in the present study are shown.

If the concentration of CO_2 declines, RuBisCO can bind oxygen molecules (O_2) in place of CO_2. This process is called photorespiration which does not generate ATP. See Campbell et al. [1] for more details.

Sucrose is synthesized in cytoplasm from TPs coming from chloroplast. In apoplastic phloem loaders, sucrose/glucose diffuse into the apoplast, mediated by SWEET transporter proteins located at the cell plasma membrane [6], and then actively transported into sieve elements via SUT1 sucrose transporter [7,8]. In the appoplast (and also within cells), invertase enzymes hydrolyse sucrose into glucose and fructose where they can be accessible by microorganisms present at the intercellular spaces of the leaf tissue [9].

Bacteria

The underlying pathways related to transport and growth of bacteria (*i.e.*, *E. coli*) are shown in Fig. B. Note that these detailed subcellular pathways are shown here only to provide an idea about the underlying events that lead to bacterial behaviors (motility, chemotaxis, AI-2 synthesis and glucose uptake) that have been included in the modeling framework presented in this manuscript. Bacterial motility (Fig. Ba) is referred to as the random tumbling motion of a bacterium cell due to rapid changes in the direction of flagellar rotation. At the cytoplasmic membrane of bacteria, several chemoreceptors called methyl-accepting chemotaxis proteins (MCPs) exist that are sensitive to different extracellular molecules (*i.e.*, ligands). While there is no external molecule bound to the MCPs, bacterium continues the tumbling motion. If an external attractant molecule binds to the MCPs (Fig. Bb), bacterium continues to swim toward the attractive chemicals (e.g., glucose, AI-2, O_2) [10]. Bacteria communicate by producing and sensing signaling molecules. The intercellular signaling known as quorum sensing (QS) allows bacteria to regulate production of gene products, such as enzymes, and coordinate behavioral responses at high cell densities [14]. Among various QS systems discovered in *E. coli* [15], auto-inducer 2 (AI-2) signaling pathway is considered here (Fig. Bc). AI-2 has a key role in quorum sensing and biofilm formation in *E. coli*. AI-2 is a chemoattractant for *E coli* in a process mediated by LsrB binding protein and Tsr chemoreceptor

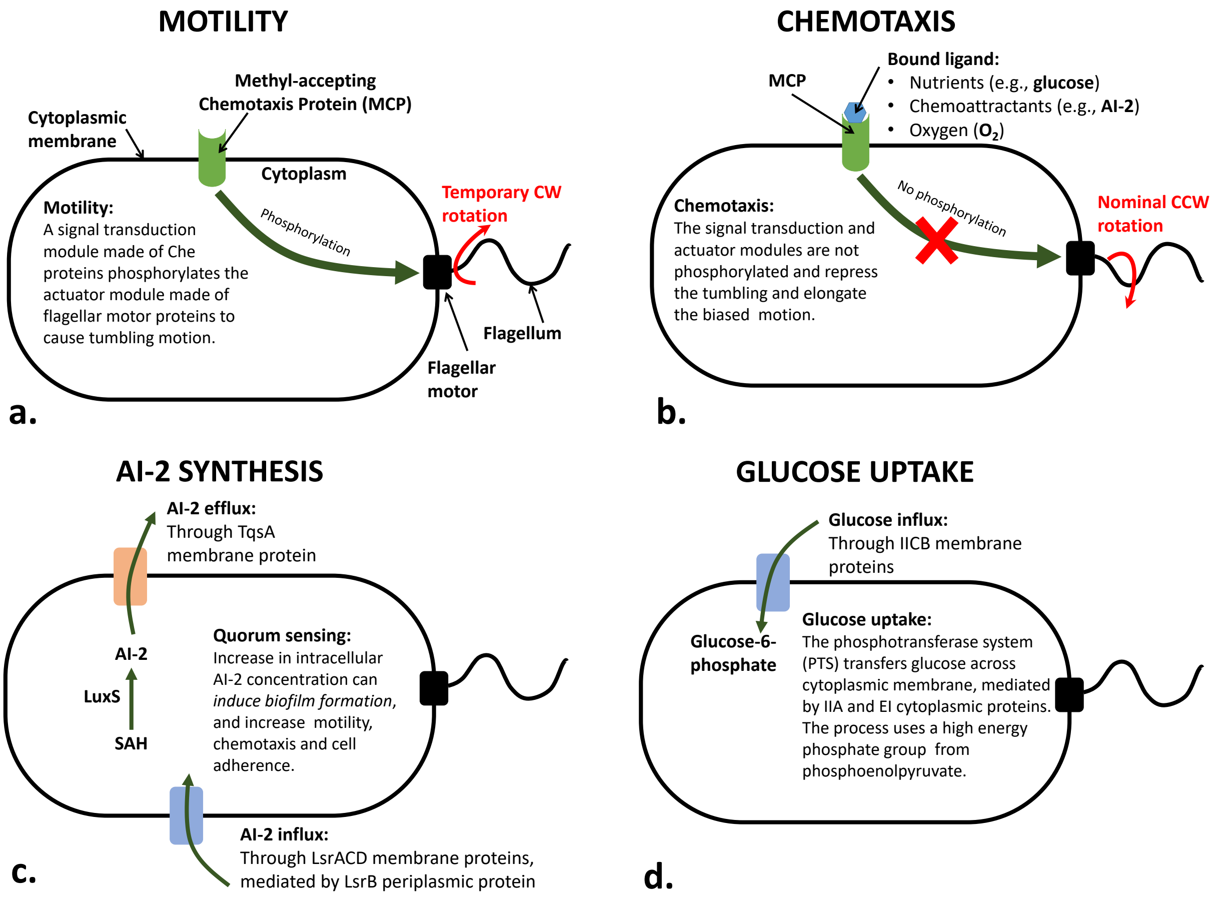

Figure B: A brief illustration of the underlying pathways leading to a) bacterial cell motility [10], b) chemotaxis [10,11], c) AI-2 synthesis [12], and d) glucose uptake by bacteria [13].

[12]. It enhances bacterial chemotaxis toward external attractants [11] like glucose and oxygen.

Bacterial growth is often limited by availability of nutrients. In *E. coli*, the growth limiting nutrients [16] can be sources of carbon (*e.g.*, glucose), nitrogen (e.g., ammonium), phosphorus (inorganic phosphate), etc. Oxygen can also affect the growth of *E. coli* as a facultative anaerobe [17]. Most of the microorganisms, including *E. coli*, prefer glucose as their primary carbon source [18]. If sufficient glucose is present in the growth medium, synthesis of the enzymes needed for transport and metabolism of the less favorable sugars will be repressed. This phenomena is called carbon catabolite repression (CCR) [13]. Glucose (and many other carbohydrates) is assimilated by bacteria through the phosphotransferase system (PTS) (Fig. Bd) [17].

Model overview

An overview of the model, including all involved species and their interconnections are shown in Fig. C.

Derivation of transport equations for $\mathrm{CO_2}$ and $\mathrm{O_2}$

Transport of species, i, (*i.e.*, CO_2 and O_2) in the gas and water phases are governed by:

$$S_g\phi\frac{\partial c_{i,g}}{\partial t} = \nabla\cdot(S_g\phi D_{i,g}\nabla c_{i,g}) \tag{S1}$$

$$(S_{wf}+S_{wb})\phi\frac{\partial c_{i,w}}{\partial t} = \nabla\cdot((S_{wf}+S_{wb})\phi D_{i,w}\nabla c_{i,w}) + (S_{wf}+S_{wb})\phi R_{i,w} \tag{S2}$$

where $R_{i,w}$ is an arbitrary source term ($\mathrm{mol/m^3\cdot s}$). Assuming the equilibrium between gas and water phases to be described by Henry's law [19]:

$$c_{i,w} = RTK_{H,i}c_{i,g} \tag{S3}$$

and plugging into Eq. 4, the total concentration of each species in the REV is defined as:

$$c_i = \left(S_g + (S_{wf} + S_{wb})RTK_{H,i}\right)\phi c_{i,g} \tag{S4}$$

Using Eq. S4, Eq. S2 can be rewritten as:

$$\begin{aligned}\left(S_g + (S_{wf} + S_{wb})RTK_{H,i}\right)\phi\frac{\partial c_{i,g}}{\partial t} = \nabla\cdot\left((S_g D_{i,g} + (S_{wf} + S_{wb})D_{i,w}RTK_{H,i})\phi\nabla c_{i,g}\right)\\ +(S_{wf} + S_{wb})\phi R_{i,w}\end{aligned} \tag{S5}$$

Finally, by adding Eq. S1 and Eq. S5 and applying Eq. S4, the combined transport equation in the REV is obtained as:

$$\frac{\partial c_i}{\partial t} = \nabla\cdot(D_{i,\mathit{eff}}\nabla c_i) + (S_{wf} + S_{wb})\phi R_{i,w} \tag{S6}$$

where $D_{i,\mathit{eff}}$ is effective diffusivity of species i in the porous media:

$$D_{i,\mathit{eff}} = \frac{S_g D_{i,g} + (S_{wf} + S_{wb})D_{i,w}RTK_{H,i}}{S_g + (S_{wf} + S_{wb})RTK_{H,i}} \tag{S7}$$

Input parameters of the model

Input data for the simulations are shown in Table A. Details of some of the input parameters are discussed here.

CO_2 assimilation parameters

RuBisCO-limited: Temperature dependence of kinetics parameters of RuBisCO (Eq. 16) including $V_{c,max}$, K_{m,co_2} and K_{m,o_2} can be described by Arrhenius equations [45]. The activation energy for the maximum carboxilation capacity of RuBisCO, $V_{c,max}$, in spinach leaves was $64900\ \mathrm{J/mol}$. Based on the data of Yamori et al. [46] at 25°C, the maximum carboxilation capacity $(\mathrm{mol/m^3 \cdot s})$ was calculated as:

$$V_{c,max} = 49.9 \times 10^{-6} \exp\left(\frac{64900(T-298)}{298RT}\right) \alpha_t \tag{S8}$$

Here, α_t is the reciprocal of the leaf thickness $(1/\mathrm{m})$, which is used to get a volumetric value for $V_{c,max}$. The temperature dependence of the Michaelis-Menten constants of RuBisCO (Pa), within a range of 5-40 °C, were calculated as [47]:

$$K_{m,co_2} = 27 \exp\left(\frac{80990(T-298)}{298RT}\right) \tag{S9}$$

$$K_{m,o_2} = 16500 \exp\left(\frac{23720(T-298)}{298RT}\right) \tag{S10}$$

The CO_2 compensation point without dark respiration (Pa), $\mathit{\Gamma}^*$, is defined as the partial pressure of CO_2 at which no net assimilations occur [47]. The temperature dependence of $\mathit{\Gamma}^*$ was reported by Medlyn et al. [48] for spinach leaves:

$$\mathit{\Gamma}^* = 0.101325\left(42.7 + 1.68(T-298.15) + 0.0012(T-298.15)^2\right) \tag{S11}$$

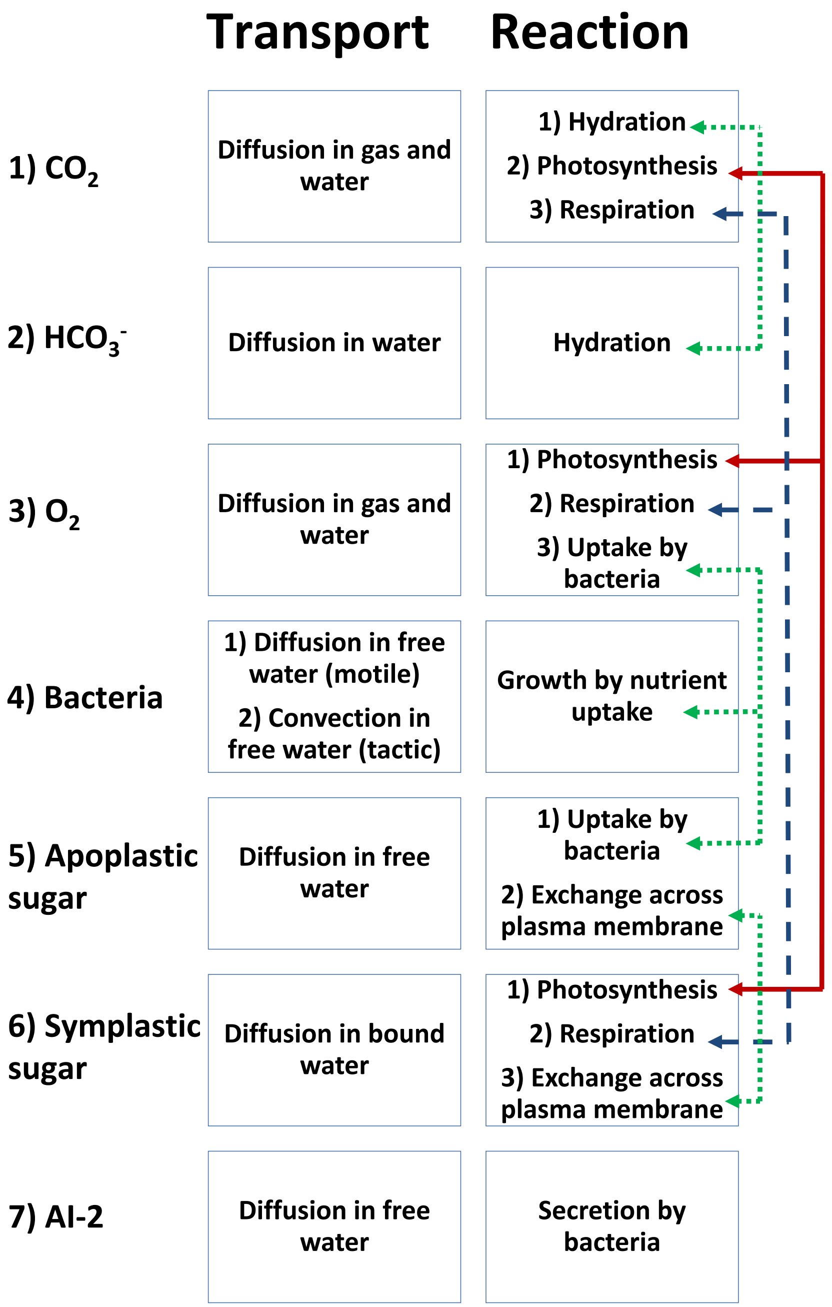

Figure C: A big picture of the model showing the interconnection between all species via reaction terms. Note that free water refers to the intercellular water, and bound water refers to intracellular water.

Electron transport-limited: The volumetric rate of electron transport (in Eq. 17) $(\mathrm{mol/m^3.s})$, J, can be described in terms of light limited, J_{ll}, and light saturated, J_{ls}, rates of electron transport in PS II [49]:

$$J = \frac{J_{ll} + J_{ls} - \sqrt{(J_{ll} + J_{ls})^2 - 4\theta J_{ll} J_{ls}}}{2\theta}\alpha_t \tag{S12}$$

where $\theta = 0.97$. The light limited rate of electron transport $(\mathrm{mol/m^2 \cdot s})$, J_{ll}, is determined from the amount of the available light $(\mathrm{mol/m^2 \cdot s})$, I, to be absorbed by the chlorophyll pigments that can vary by the light wavelength [20]:

$$J_{ll} = \alpha_{PSII} \Phi_{PSII} I \tag{S13}$$

where $\alpha_{PSII} = 0.5$ is the fraction of absorbed photons driving PS II electron transport, and $\Phi_{PSII} = 0.85$ mol/mol is the maximum quantum efficiency of PS II in electron transport. The distribution of light within the leaf tissue was calculated using Beer-Lambert's law:

$$\frac{\partial I}{\partial z} = a_{chl} I \tag{S14}$$

where a_{chl} is the absorption coefficient of chlorophyll *a* which depends on the specific absorption $(\mathrm{m^2/mol})$ [20,34], a^*_{chl}, and the density of chlorophyll *a* within the leaf tissue $(\mathrm{mol/m^3})$, ρ_{chl}:

$$a_{chl} = a^*_{chl} \rho_{chl} \tag{S15}$$

The profile of chlorophyll density within spinach leaves was obtained from Vogelmann and Evans [20] who measured the chlorophyll fluorescence profiles within spinach leaves (Fig. Da).

The light saturated rate of electron transport $(\mathrm{mol/m^2 \cdot s})$ is defined as:

$$J_{ls} = \beta J_{max} \tag{S16}$$

where β is defined here as the relative photosynthetic capacity whose profile was obtained from

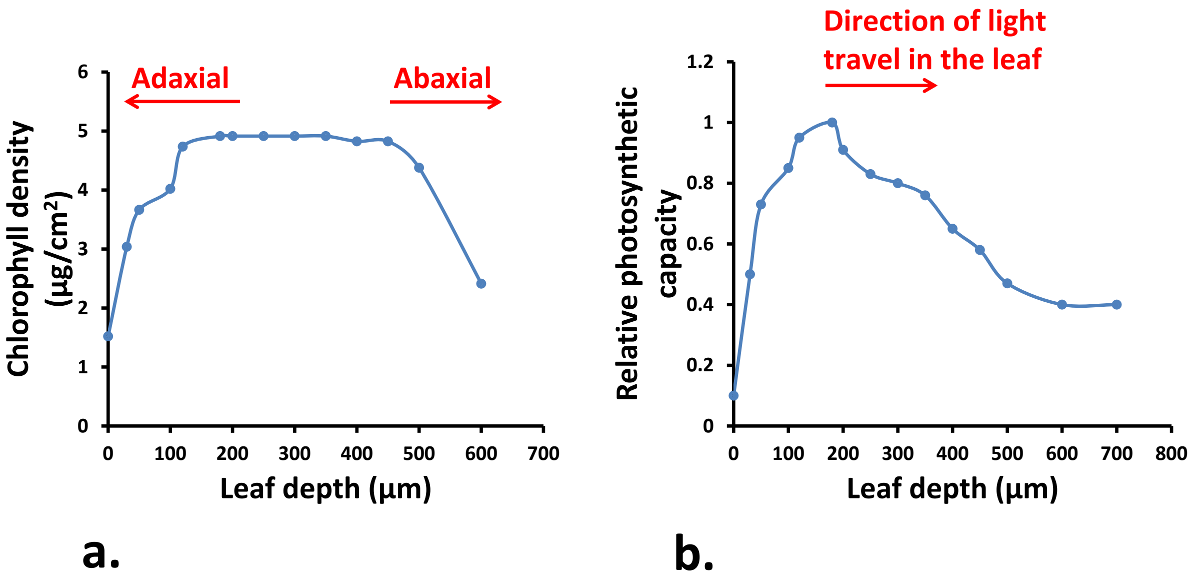

Figure D: Profiles of a) chlorophyll density and b) relative photosynthetic capacity within spinach leaves, inspired from Vogelmann and Evans [20] and Evans and Vogelman [50], respectively.

Evans and Vogelman [20] (Fig. Db), and J_{max} is the maximum rate of electron transport $(\mathrm{mol/m^2} \cdot \mathrm{s})$, and can be calculated by [51]:

$$J_{max} = 1 \times 10^6 \frac{\exp\left(37.08 - \frac{79500}{RT}\right)}{1 + \exp\left(\frac{650T - 201000}{RT}\right)} \tag{S17}$$

TPU-limited: A value of $9.19 \times 10^{-6} \mathrm{mol/m^2 \cdot s}$ was adopted for the TPU rate (in Eq. 18) $(\mathrm{mol/m^2} \cdot \mathrm{s})$, T_p [19]. So, the volumetric value of TPU rate is:

$$T_p^* = T_p \alpha_t \tag{S18}$$

Table A: Input Parameters

Parameter	Symbol	Value	Units	Source
Dimensions				
Typical values for stomatal size (equivalent diameter), when it is not specified	D_{stoma}	4×10^{-6}, in dark	m	Assumed from measurements
		16×10^{-6}, in blue light	m	Assumed from measurements
		14×10^{-6}, in white light	m	Assumed from measurements
		12×10^{-6}, in red light	m	Assumed from measurements
		9×10^{-6}, in green light	m	Assumed from measurements
Depth of leaf section	H_{leaf}	700×10^{-6}	m	Vogelmann and Evans [20]
Depth of spongy mesophyll layer	H_{spg}	370×10^{-6}	m	Assumed
Depth of palisade mesophyll layer	H_{pls}	270×10^{-6}	m	Assumed
Depth of epidermis layer	H_{stoma}	30×10^{-6}	m	Assumed
Width of the domain	W_{leaf}	80×10^{-6}	m	Assumed
Density				
Carbon dioxide	ρ_{co_2}	Ideal gas	$\mathrm{kg/m^3}$	Assumed
Oxygen	ρ_{o_2}	Ideal gas	$\mathrm{kg/m^3}$	Assumed

Diffusivity				
Motile-only bacteria in water	$\eta_{bac,mot}$	1×10^{-12}	m^2/s	Wu et al. [21]
Wild type bacteria in water	$\eta_{bac,wt}$	5×10^{-11}	m^2/s	Wu et al. [21]
Carbon dioxide in air	$D_{co_2,g}$	1.6×10^{-5}	m^2/s	Lide [22]
Oxygen in air	$D_{o_2,g}$	1.6×10^{-5}	m^2/s	Lide [22]
Carbon dioxide in water	$D_{co_2,w}$	1.67×10^{-9}	m^2/s	Lide [22]
Oxygen in water	$D_{o_2,w}$	2.01×10^{-9}	m^2/s	Lide [22]
HCO_3^- in water	$D_{HCO_3^-,w}$	1.17×10^{-9}	m^2/s	Geers and Gros [23]
Glucose in water	$D_{gluc,w}$	0.67×10^{-9}	m^2/s	Nobel [24]
Sucrose in water	$D_{suc,w}$	0.52×10^{-9}	m^2/s	Nobel [24]
AI-2 in water	$D_{AI2,w}$	1×10^{-10}	m^2/s	Assumed from Stewart [25]
Leaf properties				
Porosity	ϕ	0.95	m^3/m^3	Ranjbaran and Datta [26]
Specific surface area of porous zone	α_p	1×10^5	m^2/m^3	Calculated from Rahman [27]
SWEET transporters				
Permeability, glucose	P_{gluc}	1.34×10^{-1}	m/s	Calculated
Permeability, sucrose	P_{suc}	1.04×10^{-1}	m/s	Calculated
Population density on plasma membrane	ρ_{SWEET}	1×10^{12}	$1/m^2$	Assumed from Nobel [24]
Cross section area	A_{SWEET}	1×10^{-19}	m^2	Calculated from Carruthers [28], and Dend and Yan [29]

Half saturation constant	K_{SWEET}	9.1	mol/m^3	Assumed from Chen et al. [30]
Saturations				
Spongy mesophyll, gas	$S_{g,spg}$	0.41	m^3/m^3	Ranjbaran and Datta [26]
Spongy mesophyll, free water	$S_{fw,spg}$	0.15	m^3/m^3	Ranjbaran and Datta [26]
Spongy mesophyll, bound water	$S_{bw,spg}$	0.44	m^3/m^3	Ranjbaran and Datta [26]
Palisade mesophyll, gas	$S_{g,pls}$	0.20	m^3/m^3	Ranjbaran and Datta [26]
Palisade mesophyll, free water	$S_{fw,pls}$	0.15	m^3/m^3	Ranjbaran and Datta [26]
Palisade mesophyll, bound water	$S_{bw,pls}$	0.65	m^3/m^3	Ranjbaran and Datta [26]
Epidermis layer, gas	$S_{g,epd}$	0.20	m^3/m^3	Assumed
Epidermis layer, free water	$S_{fw,epd}$	0.15	m^3/m^3	Assumed
Epidermis layer, bound water	$S_{bw,epd}$	0.65	m^3/m^3	Assumed
Stomatal cavity, gas	$S_{g,stm}$	0.90	m^3/m^3	Assumed
Stomatal cavity, free water	$S_{fw,stm}$	0.10	m^3/m^3	Assumed
Sub-saturation coefficients				
Chloroplast, mesophyll	$\gamma_{chl,mes}$	0.254	m^3/m^3	Winter et al. [31]
Chloroplast, guard cell	$\gamma_{chl,grd}$	0.254	m^3/m^3	Assumed
Chloroplast, epidermis	$\gamma_{chl,epd}$	0	m^3/m^3	Assumed
Mitochondria	γ_{mit}	0.0082	m^3/m^3	Winter et al. [31]

Gas solubility in water				
Henry's constant for CO_2, at 20 °C	K_{H,co_2}	0.3876×10^{-3}	$\mathrm{mol/m^3 \cdot Pa}$	Lide [22]
Henry's constant for O_2, at 20 °C	K_{H,o_2}	0.0137×10^{-3}	$\mathrm{mol/m^3 \cdot Pa}$	Lide [22]
Carbon dioxide hydration				
Reaction constant 1	k_1	0.039	1/s	Jolly [32]
Reaction constant 2	k_2	23	1/s	Jolly [32]
Acid dissociation constant for H_2CO_3	K_{hyd}	0.25	$\mathrm{mol/m^3}$	Jolly [32]
pH	pH	7.0, Water<br>6.5, Leaf		Assumed<br>Assumed from Babic and Watada [33]
Light absorption by chloroplasts				
Specific absorption, Blue light	$a^*_{chl,blu}$	2600	$\mathrm{m^2/mol}$	Assumed from Vogelmann and Evans [20]
Specific absorption, Green light	$a^*_{chl,grn}$	1500	$\mathrm{m^2/mol}$	Assumed from Vogelmann and Evans [20]
Specific absorption, Red light	$a^*_{chl,red}$	2000	$\mathrm{m^2/mol}$	Assumed from Mitchell and Kiefer [34] and Vogelmann and Evans [20]
Specific absorption, white light	$a^*_{chl,wht}$	2100	$\mathrm{m^2/mol}$	Assumed

Nutrient uptake by bacteria				
Yield of glucose on bacteria	$Y_{gluc/bac}$	9.4×10^{-15}	mol/cell	Assumed from Kayser et al. [35]
Yield of oxygen on bacteria	$Y_{o_2/bac}$	2.9×10^{-14}	mol/cell	Assumed from Shiloach and Fass [36]
AI-2 reaction				
Synthesis rate constant	$k_{1,AI2}$	1×10^{-24}, no glucose	$\mathrm{mol/cell \cdot s}$	Li et al. [37], Xu et al. [38] and Wang et al. [39]
		1×10^{-23}, with glucose	$\mathrm{mol/cell \cdot s}$	
Bacterial chemotaxis				
Chemotactic sensitivity coefficient, *E. coli*	χ_0	10×10^{-9}	$\mathrm{m^2/s}$	Ford et al. [40]
Receptor-ligand dissociation constant, glucose	$K_{d,gluc}$	0.1	$\mathrm{mol/m^3}$	Ford and Lauffenburger [41]
Receptor-ligand dissociation constant, oxygen	$K_{d,o2}$	0.013	$\mathrm{mol/m^3}$	Delgado-Nixon et al. [42]
Receptor-ligand dissociation constant, AI-2	$K_{d,AI2}$	0.007	$\mathrm{mol/m^3}$	Assumed from Jani et al. [12]
Bacteria growth				
Maximum growth rate constant, at 25 °C	$\zeta_{max,gr}$	1.00×10^{-4}	1/s	Assumed from Kovarova et al. [43]
Monod half saturation constant, glucose	K_{gluc}	184.8×10^{-6}	$\mathrm{mol/m^3}$	Kovarova et al. [43]

Monod half saturation constant, Oxygen	K_{o_2}	121×10^{-6}	$\mathrm{mol/m^3}$	Stolper et al. [44]

Experimental procedure

Bacterial strains and inoculum preparation

A loop of frozen (at -80 °C) culture of *E. coli* cells (wild type ampicillin-resistant K-12 MG1655 carrying pUC19 plasmid, or incessantly motile (but not chemotactic) kanamycin-resistant K-12 BW25113 (Δ *CheZ*)) were grown in sterile LB broth (a 25 $\mathrm{g/l}$ of LB powder dissolved in Milli-Q water), supplemented with 100 $\mu\mathrm{g/ml}$ ampicillin or 30 $\mu\mathrm{g/ml}$ kanamycin, in a shaker incubator at 37 °C and 220 rpm. This was followed by a second overnight incubation in fresh LB broth. The bacterial culture were then harvested by two successive centrifugation steps (Sorrvall legend RT+centrifuge, Thermo Scientific, USA) at 2700 g for 10 min to efficiently remove the LB broth. The cell pellets were resuspended in sterile 0.85% NaCl (saline) solution and the concentration of final bacterial populations was adjusted to $\sim 10^8$ $\mathrm{CFU/ml}$ by using spectrophotometry analysis (Agilent Technologies, Inc., Mattapoiset, MA).

Leaf inoculation and light exposure

Experimental procedure to determine total amount of infiltration is shown in Fig. E. Fresh baby spinach leaves were purchased at a local retail store. Samples of 5 g containing about 8 leaves were used. The leaves were placed in sterile petri dishes with cover lids and were kept at room temperature for about 45 min under the illumination condition that was under study. This was done to make sure that the leaf microstructures were in equilibrium with the illumination condition. Then the samples were spot inoculated with 500 μl of *E. coli* cell suspension to reach an initial population of $\sim 3 \times 10^7$ $\mathrm{CFU/g}$. The inoculum was then gently spread on the leaf surface using a sterile loop to uniformly cover the surface. The lid of petri dishes were placed to avoid evaporation

of the inoculum at the leaf surface. The samples were exposed to white/blue/red/green light with intensity of 100 $\mu\text{mol}/\text{m}^2{\cdot}\text{s}$, or kept in the dark. All exposures were performed at room temperature for 2 h. The light intensity was measured by a digital luxmeter (PM6612, Peak Meter ®, China) with $\pm 3\%$ accuracy. The illumination chamber was shielded thoroughly to make sure that the samples would only receive the expected wavelengths.

Bacterial infiltration assay

After exposure treatment, the surface of the leaves was washed by sterile 0.85% NaCl (saline) solution and left to dry. Then, they were sprayed with 70% ethanol in two successive steps to remove any surface bacteria. The surface sterilizatrion was examined (Fig. F) by gently pushing the surface of some treated leaves on LB agar plates and incubate them. The surface sanitation was considered effective when more than 99.99999% of inoculated bacteria at the surface were inactivated (about 8-log CFU/ml reduction).

The surface sterilized leaves of each sample were crushed in a sterile bowl and was added with 45 ml sterile 0.85% NaCl (saline) solution. The homogenized sample was serially diluted in sterile 0.85% NaCl (saline) solution and surface plated onto LB agar containing 100 $\mu\text{g}/\text{ml}$ ampicillin or 30 $\mu\text{g}/\text{ml}$ kanamycin. To make sure that the natural microbiota on the leaf surface were not growing in the growth medium containing ampicllin or kanamycin, control samples without inoculation were also homogenized and plated. The inoculated petri dishes were incubated at 30°C for 24 h and the colonies were enumerated to find the bacterial count. No growth was observed in any of the uninoculated plates (Fig. G) implying that the natural microbiota were not ampicillin or kanamycin resistant and would not be counted as infiltrated bacteria.

Microscopy imaging of stomatal aperture

The microscopy imaging experiments were done using an epi-fuorescent microscope (DM5500, Leica Microsystems, Exton, PA, USA) with 20x or 63x water immersion objectives. Before microscopy, three leaves were either exposed to white/blue/red/green light with an intensity of

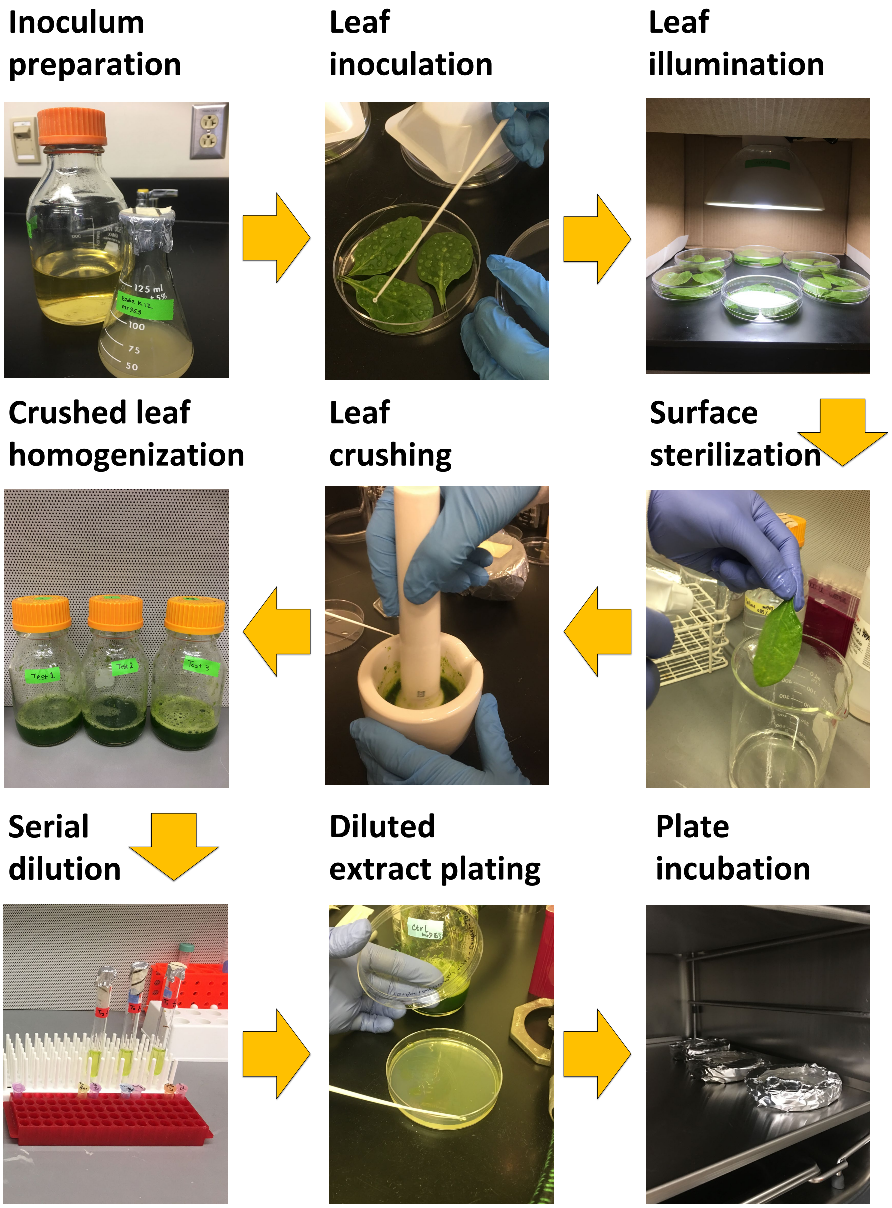

Figure E: Experimental procedure to determine total amount of infiltration.

Test 1 **Test 2** **Test 3**

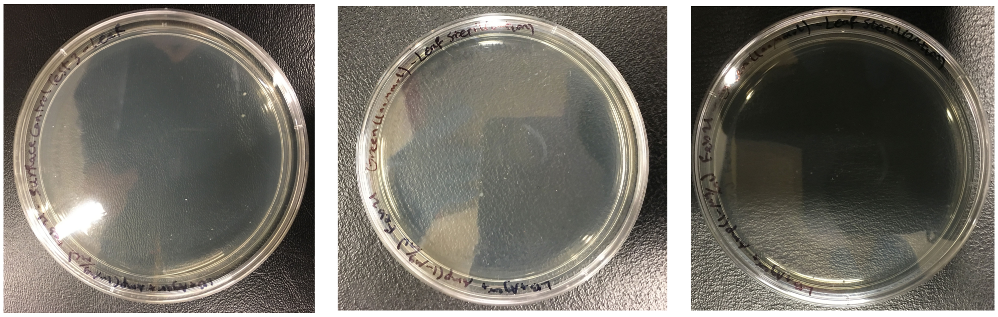

a.

Bacteria: ampicillin-resistant *E. coli* K-12 MG1655

	Illumination condition	Number of colonies detected	Dilution factor	Leaf side	Number of bacteria at the leaf surface after surface sanitation (CFU)
**Test 1**	White<br>100 (µmol/m^2.s)	3	1/1	Adaxial	3
**Test 2**	Green<br>100 (µmol/m^2.s)	1	1/1	Adaxial	1
**Test 3**	Green<br>100 (µmol/m^2.s)	0	1/1	Adaxial	0

b.

Figure F: Results of the leaf surface sanitation treatments. The inoculated surface of the leaves were washed by sterile 0.85% NaCl (saline), after 2 h illumination, and let to dry. Then, they were sprayed with 70% ethanol in two successive steps to remove any surface bacteria.

Test 1 **Test 2** **Test 3**

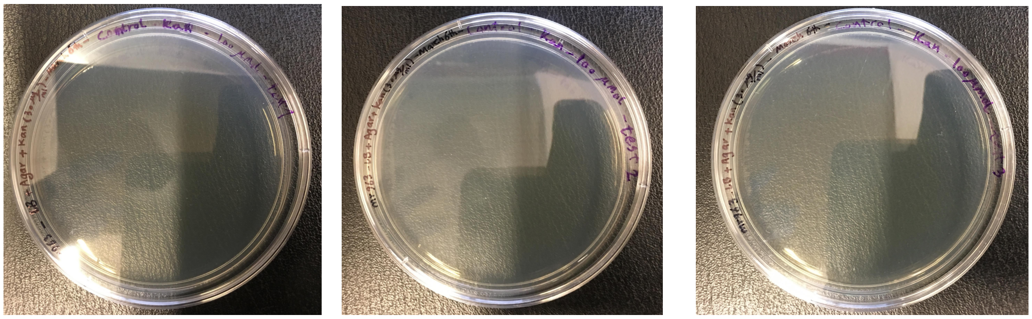

a.

No antibiotic-resistant bacteria

	Illumination condition	Antibiotic	Dilution factor	Leaf side	Number of colonies detected
**Test 1**	White<br>100 (μmol/m^2.s)	Kanamycin	1/500	Adaxial	0
**Test 2**	White<br>100 (μmol/m^2.s)	Kanamycin	1/500	Adaxial	0
**Test 3**	White<br>100 (μmol/m^2.s)	Kanamycin	1/500	Adaxial	0
**Test 4**	White<br>100 (μmol/m^2.s)	Ampicillin	1/500	Adaxial	0
**Test 5**	White<br>100 (μmol/m^2.s)	Ampicillin	1/500	Adaxial	0
**Test 6**	White<br>100 (μmol/m^2.s)	Ampicillin	1/500	Adaxial	0

b.

Figure G: Control tests to make sure the natural microbiota on the leaf surface were not growing in the growth medium containing ampicillin or kanamycin.

$100\ \mu\text{mol/m}^2 \cdot \text{s}$, or were kept in the dark for 45 min. After illumination, samples ($5\ \text{mm} \times 5\ \text{mm}$) from three arbitrary locations of each leaf were cut and immediately used for microscopy. For each experimental condition, data of stomatal aperture were gathered from more than 100 stomata. The measurements were done using ImageJ software. The results of measurements of the stomatal aperture when using various light wavelengths as well as dark condition, are shown in Fig. H.

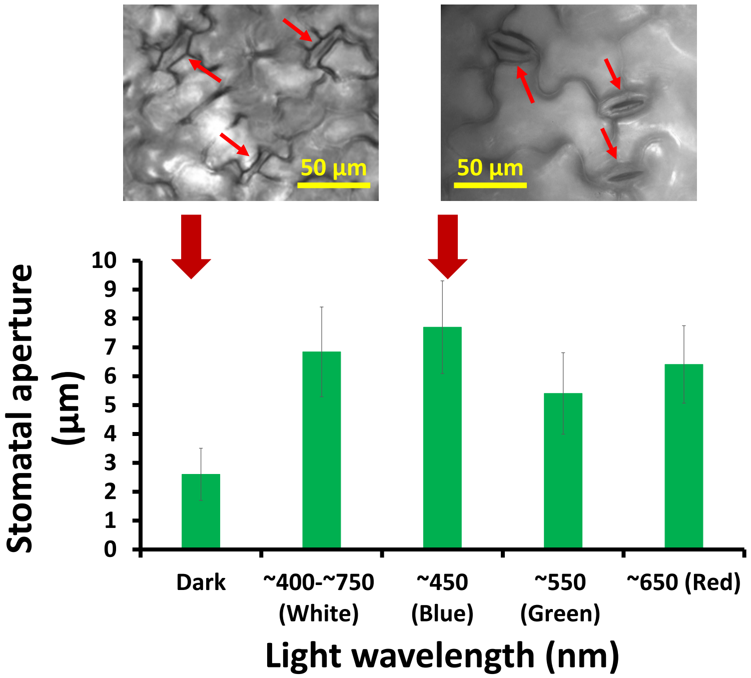

Figure H: Measured stomatal aperture of spinach leaves under different light colors of 100 $\mu\text{mol}/\text{m}^2 \cdot \text{s}$, and dark condition for 45 min. Each data presents an average of 100 measurements of the stomatal aperture. The error bars show the standard deviations. Representative confocal microscopy images related to dark condition (smallest aperture) and blue light illumination condition (widest aperture) are shown as insets. Red arrows in the inset images show the location of the stomata.

Results

Measured microbial infiltration into spinach leaves

The results of the measurement of wild-type bacterial infiltration into adaxial side of spinach leaves are shown in Fig. I for dark condition, and in Fig. J for white light, Fig. K for blue light, Fig. L for green light, and Fig. M for red light, with light intensity of 100 $\mu\text{mol}/\text{m}^2 \cdot \text{s}$. The corresponding results for infiltration of *CheZ* mutant bacteria under white light of the same intensity are shown in Fig. N. Also, the corresponding results for infiltration of wild-type bacteria under white light of the same intensity into the abaxial side of the spinach leaves are shown in Fig. O. Evidence presented here are to support data shown in Fig. 4c-4e and Fig. 5a.

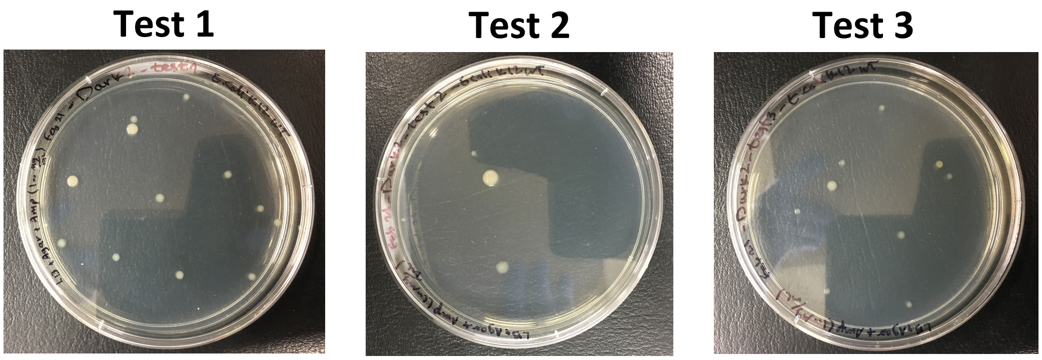

a.

Bacteria: ampicillin-resistant *E. coli* K-12 MG1655

	Illumination condition	Number of colonies detected	Dilution factor	Leaf side	Leaf sample weight (g)	Number of bacterial infiltration into the leaves (CFU/g)	Bacterial infiltration (log CFU/g)
**Test 1**	Dark	12	1/500	Adaxial	5.06	2400	3.38
**Test 2**	Dark	3	1/500	Adaxial	5.05	600	2.78
**Test 3**	Dark	9	1/500	Adaxial	5.01	1800	3.26
**Mean**						1600	3.13
**Standard deviation**						748.3	0.26

b.

Figure I: Results of the colony growth of ampicillin-resistant *E. coli* K-12 MG1655 on LB-agar medium containing 100 $\mu g/ml$ ampicillin. The inoculated leaves were kept in dark condition for 2 h.

Test 1 **Test 2** **Test 3**

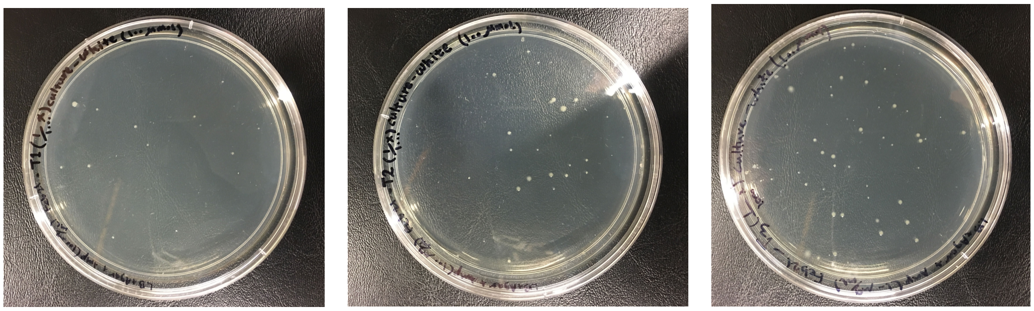

a.

Bacteria: ampicillin-resistant *E. coli* K-12 MG1655

	Illumination condition	Number of colonies detected	Dilution factor	Leaf side	Leaf sample weight (g)	Number of bacterial infiltration into the leaves (CFU/g)	Bacterial infiltration (log CFU/g)
**Test 1**	White 100 (µmol/m^2.s)	14	1/1000	Adaxial	5.015	5600	3.75
**Test 2**	White 100 (µmol/m^2.s)	39	1/1000	Adaxial	5.011	15600	4.19
**Test 3**	White 100 (µmol/m^2.s)	54	1/1000	Adaxial	5.013	21600	4.33
**Mean**						14266.7	4.09
**Standard deviation**						6599.7	0.25

b.

Figure J: Results of the colony growth of ampicillin-resistant *E. coli* K-12 MG1655 on LB-agar medium containing 100 $\mu\mathrm{g/ml}$ ampicillin. The inoculated leaves were exposed to white light, from adaxial side, with an intensity of 100 $\mu\mathrm{mol/m^2} \cdot \mathrm{s}$ for 2 h.

Test 1 Test 2 Test 3 Test 4

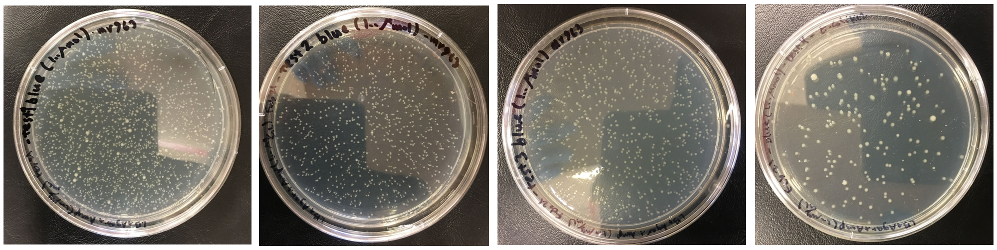

a.

Bacteria: ampicillin-resistant *E. coli* K-12 MG1655

	Illumination condition	Number of colonies detected	Dilution factor	Leaf side	Leaf sample weight (g)	Number of bacterial infiltration into the leaves (CFU/g)	Bacterial infiltration (log CFU/g)
**Test 1**	Blue 100 (μmol/m².s)	1978	1/500	Adaxial	5.012	395600	5.60
**Test 2**	Blue 100 (μmol/m².s)	887	1/500	Adaxial	5.010	177400	5.25
**Test 3**	Blue 100 (μmol/m².s)	861	1/500	Adaxial	5.021	172200	5.23
**Test 4**	Blue 100 (μmol/m².s)	170	1/500	Adaxial	5.011	34000	4.53
**Mean**						194800	5.15
**Standard deviation**						129413	0.39

b.

Figure K: Results of the colony growth of ampicillin-resistant *E. coli* K-12 MG1655 on LB-agar medium containing 100 $\mu\mathrm{g/ml}$ ampicillin. The inoculated leaves were exposed to blue light, from adaxial side, with an intensity of 100 $\mu\mathrm{mol/m^2} \cdot \mathrm{s}$ for 2 h.

Test 1 Test 2 Test 3

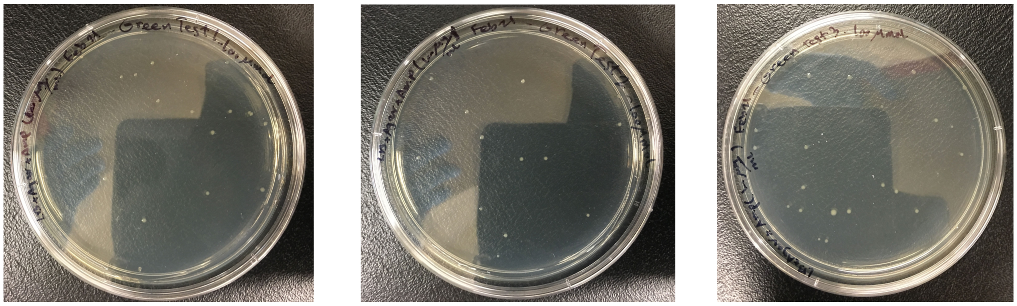

a.

Bacteria: ampicillin-resistant *E. coli* K-12 MG1655

	Illumination condition	Number of colonies detected	Dilution factor	Leaf side	Leaf sample weight (g)	Number of bacterial infiltration into the leaves (CFU/g)	Bacterial infiltration (log CFU/g)
**Test 1**	Green 100 (μmol/m².s)	13	1/500	Adaxial	5.051	2600	3.42
**Test 2**	Green 100 (μmol/m².s)	11	1/500	Adaxial	5.018	2200	3.34
**Test 3**	Green 100 (μmol/m².s)	20	1/500	Adaxial	5.008	4000	3.60
**Mean**						2933.3	3.45
**Standard deviation**						771.7	0.11

b.

Figure L: Results of the colony growth of ampicillin-resistant *E. coli* K-12 MG1655 on LB-agar medium containing 100 $\mu\mathrm{g/ml}$ ampicillin. The inoculated leaves were exposed to green light, from adaxial side, with an intensity of 100 $\mu\mathrm{mol/m^2} \cdot \mathrm{s}$ for 2 h.

Test 1 **Test 2** **Test 3**

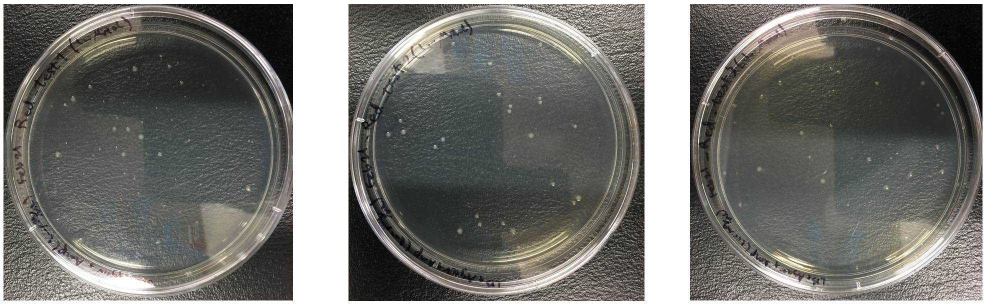

a.

Bacteria: ampicillin-resistant *E. coli* K-12 MG1655

	Illumination condition	Number of colonies detected	Dilution factor	Leaf side	Leaf sample weight (g)	Number of bacterial infiltration into the leaves (CFU/g)	Bacterial infiltration (log CFU/g)
**Test 1**	Red 100 (μmol/m^2.s)	60	1/500	Adaxial	5.010	12000	4.08
**Test 2**	Red 100 (μmol/m^2.s)	27	1/500	Adaxial	5.021	5400	3.73
**Test 3**	Red 100 (μmol/m^2.s)	32	1/500	Adaxial	5.001	6400	3.81
**Mean**						7933.3	3.87
**Standard deviation**						2904.4	0.15

b.

Figure M: Results of the colony growth of ampicillin-resistant *E. coli* K-12 MG1655 on LB-agar medium containing 100 $\mu\mathrm{g/ml}$ ampicillin. The inoculated leaves were exposed to red light, from adaxial side, with an intensity of 100 $\mu\mathrm{mol/m^2} \cdot \mathrm{s}$ for 2 h.

Test 1 **Test 2** **Test 3**

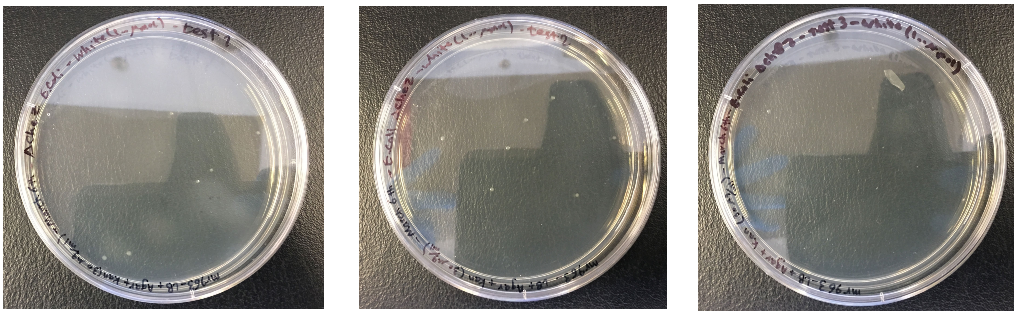

a.

Bacteria: kanamycin-resistant *E. coli* K-12 BW25113 (ΔCheZ)

	**Illumination condition**	**Number of colonies detected**	**Dilution factor**	**Leaf side**	**Leaf sample weight (g)**	**Number of bacterial infiltration into the leaves (CFU/g)**	**Bacterial infiltration (log CFU/g)**
**Test 1**	White 100 (μmol/m^2.s)	6	1/500	Adaxial	5.051	1200	3.08
**Test 2**	White 100 (μmol/m^2.s)	8	1/500	Adaxial	5.024	1600	3.20
**Test 3**	White 100 (μmol/m^2.s)	1	1/500	Adaxial	5.021	200	2.30
**Mean**						1000	2.86
**Standard deviation**						588.8	0.40

b.

Figure N: Results of the colony growth of kanamycin-resistant *E. coli* K-12 BW25113 (Δ *CheZ*) on LB-agar medium containing 30 $\mu\mathrm{g/ml}$ kanamycin. The inoculated leaves were exposed to white light, from adaxial side, with an intensity of 100 $\mu\mathrm{mol/m^2} \cdot \mathrm{s}$ for 2 h.

Test 1 Test 2 Test 3

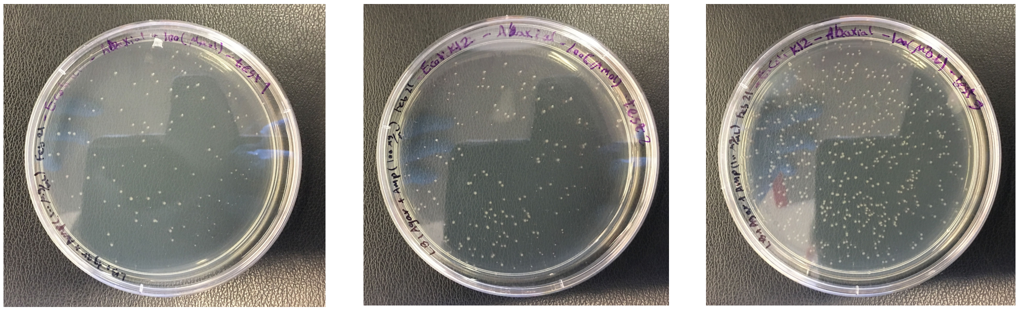

a.

Bacteria: ampicillin-resistant *E. coli* K-12 MG1655

	**Illumination condition**	**Number of colonies detected**	**Dilution factor**	**Leaf side**	**Leaf sample weight (g)**	**Number of bacterial infiltration into the leaves (CFU/g)**	**Bacterial infiltration (log CFU/g)**
**Test 1**	White 100 (μmol/m^2.s)	114	1/500	Abaxial	5.025	22800	4.36
**Test 2**	White 100 (μmol/m^2.s)	153	1/500	Abaxial	5.051	30600	4.49
**Test 3**	White 100 (μmol/m^2.s)	511	1/500	Abaxial	5.017	102200	5.01
**Mean**						51867	4.62
**Standard deviation**						35733	0.28

b.

Figure O: Results of the colony growth of ampicillin-resistant *E. coli* K-12 MG1655 on LB-agar medium containing 100 $\mu\mathrm{g/ml}$ ampicillin. The inoculated leaves were exposed to white light, from abaxial side, with an intensity of 100 $\mu\mathrm{mol/m^2} \cdot \mathrm{s}$ for 2 h.

Model predictions for bacterial flux inside stomatal cavity

Predicted bacterial flux (toward the leaf interior) within stomatal cavity after 1 h of illumination with white light intensity of 100 $\mu\mathrm{mol/m^2} \cdot \mathrm{s}$ are shown in Fig. P.

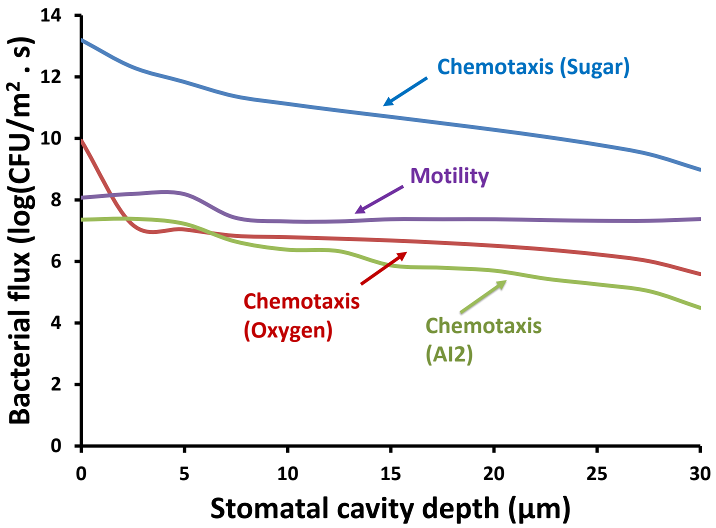

Figure P: Predicted bacterial flux (toward the leaf interior) within stomatal cavity after 1 h of illumination with white light intensity of 100 $\mu\mathrm{mol/m^2 \cdot s}$.

References

1. Campbell NA, Reece JB, Urry LA, Cain ML, Wasserman SA, Minorsky PV, et al, Biology. 8th Ed. Pearson Custom Publishing, San Francisco, CA, 2008.

2. Shimazaki KI, Doi M, Assmann SM, Kinoshita T, Light regulation of stomatal movement. Annu Rev Plant Biol, 2007; 58: 219-247.

3. Melotto M, Panchal S, Roy D, Plant innate immunity against human bacterial pathogens. Front Microbiol, 2014; 5: 411.

4. Panchal S, Chitrakar R, Thompson BK, Obulareddy N, Roy D, Hambright WS, et al, Regulation of stomatal defense by air relative humidity. Plant Physiol, 2016; 172: 2021-2032.

5. Voitsekhovskaja OV, Pakhomova MV, Syutkina AV, Gamalei YV, Heber U, Compartmentation of assimilate fluxes in leaves. II. Apoplastic sugar levels in leaves of plants with different companion cell types. Plant Biol, 2000; 2: 107-112.

6. Chen LQ, Qu XQ, Hou BH, Sosso D, Osorio S, Fernie AR, et al, Sucrose efflux mediated by SWEET proteins as a key step for phloem transport. Science, 2012; 335: 207-211.

7. Rennie EA, Turgeon R, A comprehensive picture of phloem loading strategies. PNAS, 2009; 106(33): 14162-14167.

8. Doidy J, Grace E, Kuhn Ch, Simon-Plas F, Casieri L, Wipf D, Sugar transporters in plants and in their interactions with fungi. Trends Plant Sci, 2012; 17 (7): 413-422.

9. Vargas WA, Salerno GL, The Cinderella story of sucrose hydrolysis: alkaline/neutral invertases, from cyanobacteria to unforeseen roles in plant cytosol and organelles. Plant Sci, 2010; 178: 1-8.

10. McAdams HH, Srinivasan B, Arkin AP, The evolution of genetic regulatory systems in bacteria. Nat Rev Genet, 2004; 5: 169-178.

11. Long Zh, Quaife B, Salman H, Oltvai ZN, Cell-cell communication enhances bacterial chemotaxis toward external attractants. Sci rep, 2017; 7: 12855.

12. Jani S, Seely AL, Peabody V GL, Jayaraman A, Manson MD, Chemotaxis to self-generated AI-2 promotes biofilm formation in Escherichia coli. Microbiology, 2017; 136: 1778-1790.

13. Luo Y, Zhang T, Wu H, The transport and mediation mechanisms of the common sugars in Escherichia coli. Biotechnol Adv, 2014; 32: 905-919.

14. Laganenka L, Colin R, Sourjik V, Chemotaxis towards autoinducer 2 mediates autoaggregation in Escherichia coli. Nat Commun, 2016; 7: 12984.

15. Zohar BA, Kolodkin-Gal I, Quorum Sensing in Escheichia coli: Interkingdom, Inter- and Intraspecies Dialogues, and a Suicide-Inducing Peptide. In, Kalia VC, (Ed.), Quorum Sensing vs Quorum Quenching: A Battle with No End in Sight. Springer, India, 2015.

16. Peterson CN, Mandel MJ, Silhavy TJ, Escherichia coli Starvation Diets: Essential Nutrients Weigh in Distinctly. J Biotechnol, 2005; 187(22): 7549-7553.

17. McDaniel LE, Bailey EG, Zimmerli A, Effect of oxygen supply rates on growth of Escherichia coli, I. Studies in unbaffled and baffled shake flasks. Appl Microbiol, 1965; 13(1): 109-114.

18. Gorke B, Stulke J, Carbon catabolite repression in bacteria: many ways to make the most out of nutrients. Nat Rev Microbiol, 2008; 6: 613-624.

19. Ho QT, Berghuijs HNC, Watté R, Verboven P, Herremans E, Yin X, et al, Three-dimensional microscale modelling of CO_2 transport and light propagation in tomato leaves enlightens photosynthesis. Plant Cell Environ, 2016; 39: 50-61.

20. Vogelmann TC, Evans JR, Profiles of light absorption and chlorophyll within spinach leaves from chlorophyll fluorescence. Plant Cell Environ, 2002; 25: 1313-1323.

21. Wu M, Roberts JW, Kim S, Koch DL, DeLisa MP, Collective Bacterial Dynamics Revealed Using a Three-Dimensional Population-Scale Defocused Particle Tracking Technique. Appl Environ Microbiol, 2006; 72(7): 4987-4994.

22. Lide DR, In: Handbook of Chemistry and Physics. CRC Press, Boca Raton, 1999.

23. Geers C, Gros G, Carbon dioxide transport and carbonic anhydrase in blood and muscle. Physiol Rev, 2000; 80(2): 681-715.

24. Nobel PS, Physicochenichal And Environmental Plant Physiology. 3rd Ed. Elsevier Academic Press. Burlington, MA, 2005.

25. Stewart PS, Diffusion in Biofilms. J Biotechnol, 2003; 185(5): 1485-1491.

26. Ranjbaran M, Datta AK, Pressure-driven infiltration of water and bacteria into plant leaves during vacuum cooling: A mechanistic model. J Food Eng, 2019; 246: 209-223.

27. Rahman MS, Mass-Volume-Area-Related Properties of Foods. In, Rao MA, Rizvi SSH, Datta AK (Eds). Engineering Properties of Foods. 3rd Ed., CRC Press, Boca Raton, FL, 2005.

28. Carruthers A, Facilitated Diffusion of Glucose. Physiol Rev, 1990; 70(4): 1135-1176.

29. Deng D, Yan N, GLUT, SGLT, and SWEET: Structural and mechanistic investigations of the glucose transporters. Protein Sci, 2016; 25: 546-558.

30. Chen LQ, Hou BH, Lalonde S, Takanaga H, Hartung ML, Qu XQ, et al, Sugar transporters for intercellular exchange and nutrition of pathogens. Nature, 2010; 468: 527-534.

31. Winter H, Robinson DG, Heldt HW, Subcellular volumes and metabolite concentrations in spinach leaves. Planta, 1994; 193: 530-535.

32. Jolly WL, Modern Inorganic Chemistry. McGraw-Hill, New York, NY, USA, 1985.

33. Babic I, Watada AE, Microbial populations of fresh-cut spinach leaves affected by controlled atmospheres. Postharvest Biol Tech, 1996; 9: 187-193.

34. Mitchell BG, Kiefer DA, Chlorophyll a specific absorption and fluorescence excitation spectra for fight-limited phytoplankton. Deep-Sea Res, 1988; 35(5): 639-663.

35. Kayser A, Weber J, Hecht V, Rinas U, Metabolic flux analysis of Escherichia coli in glucose-limited continuous culture. i. growth-rate-dependent metabolic efficiency at steady state. Microbiology, 2005; 151(3): 693-706.

36. Shiloach J, Fass R, Growing E. coli to high cell density historical perspective on method development. Biotechnol adv, 2005; 23(5): 345-357.

37. Li J, Wang L, Hashimoto Y, Tsao CY, Wood TK, Valdes JJ, et al, A stochastic model of Escherichia coli AI-2 quorum signal circuit reveals alternative synthesis pathways. Mol Syst Biol, 2006; 67.

38. Xu F, Song X, Cai P, Sheng G, Yu H, Quantitative determination of AI-2 quorum-sensing signal of bacteria using high performance liquid chromatography–tandem mass spectrometry. J Environ Sci, 2017; 52: 204-209.

39. Wang L, Hashimoto Y, Tsao CY, Valdes JJ, Bentley WE, Cyclic AMP (cAMP) and cAMP Receptor Protein Influence both Synthesis and Uptake of Extracellular Autoinducer 2 in Escherichia coli. J Biotechnol, 2005; 187(6): 2066-2076.

40. Ford RM, Phillips BR, Quinn JA, Lauffenburger DA, Measurement of Bacterial Random Motility and Chemotaxis Coefficients: I. Stopped-Flow Diffusion Chamber Assay. Biotechnol Bioeng, 1991; 37(7): 647-660.

41. Ford R, Lauffenburger D, Measurement of Bacterial Random Motility and Chemotaxis Coefficients: II. Application of Single-Cell-Based Mathematical Model. Biotechnol Bioeng, 1991; 37: 661-672.

42. Delgado-Nixon VM, Gonzalez G, Gilles-Gonzalez MA, Dos, a Heme-Binding PAS Protein from Escherichia coli, Is a Direct Oxygen Sensor. Biochemistry, 2000; 39: 2685-2691.

43. Kovarova K, Zehder AJB, Egli T, Temperature-Dependent Growth Kinetics of Escherichia coli ML 30 in Glucose-Limited Continuous Culture. J Bacteriol, 1996; 187(15): 4530-4539.

44. Stolper DA, Revsbech NP, Canfield DE, Aerobic growth at nanomolar oxygen concentrations. PNAS, 2010; 107(44): 18755-18760.

45. Farquhar GD, von Caemmerer S, Berry JA, A biochemical model of photosynthesis CO_2 assimilation in leaves of C_3 species. Planta, 1980; 149: 78-90.

46. Yamori W, Noguchi K, Kashino Y, Terashima I, The role of electron transport in determining the temperature dependence of the photosynthetic rate in spinach leaves grown at contrasting temperatures. Plant Cell Physiol, 2008; 49: 583-591.

47. Yin X, Struik PC, C_3 and C_4 photosynthesis models: An overview from the perspective of crop modelling. NJAS-Wagen Jo Life Sc, 2009; 57: 27-38.

48. Medlyn BE, Dreyer E, Ellsworth D, Fortreuter M, Harley PC, Kirschbaum MUF, et al, Temperature response of parameters of a biochemically based model of photosynthesis. II. A review of experimental data. Plant Cell Environ, 2002; 25: 1167–1179.

49. Buckley TN, Farquhar GD, A new analytical model for whole-leaf potential electron transport rate. Plant Cell Environ, 2004; 27: 1487-1502.

50. Evans JR, Vogelmann TC, Profiles of ^{14}C fixation through spinach leaves in relation to light absorption and photosynthetic capacity. Plant Cell Environ, 2003; 26: 547-560.

51. Harley PC, Thomas RB, Reynolds JF, Strain BR, Modelling photosynthesis of cotton grown in elevated CO_2. Plant Cell Environ, 1992; 15: 271-282.
